# Amelioration of morphine withdrawal syndrome by systemic and intranasal administration of mesenchymal stem cell‐derived secretome in preclinical models of morphine dependence

**DOI:** 10.1111/cns.14517

**Published:** 2023-11-06

**Authors:** Mauricio Quezada, Carolina Ponce, Pablo Berríos‐Cárcamo, Daniela Santapau, Javiera Gallardo, Cristian De Gregorio, María Elena Quintanilla, Paola Morales, Marcelo Ezquer, Mario Herrera‐Marschitz, Yedy Israel, Paula Andrés‐Herrera, Lucia Hipólito, Fernando Ezquer

**Affiliations:** ^1^ Center for Regenerative Medicine, Faculty of Medicine Clínica Alemana‐Universidad del Desarrollo Santiago Chile; ^2^ Department of Neuroscience, Faculty of Medicine Universidad de Chile Santiago Chile; ^3^ Molecular and Clinical Pharmacology Program, Institute of Biomedical Science, Faculty of Medicine Universidad de Chile Santiago Chile; ^4^ Department of Pharmacy and Pharmaceutical Technology and Parasitology University of Valencia Valencia Spain; ^5^ University Institute of Biotechnology and Biomedicine (BIOTECMED) University of Valencia Valencia Spain; ^6^ Research Center for the Development of Novel Therapeutic Alternatives for Alcohol Use Disorders Santiago Chile

**Keywords:** mesenchymal stem cells, neuroinflammation, opiod addiction, secretome, withdrawal

## Abstract

**Background:**

Morphine is an opiate commonly used in the treatment of moderate to severe pain. However, prolonged administration can lead to physical dependence and strong withdrawal symptoms upon cessation of morphine use. These symptoms can include anxiety, irritability, increased heart rate, and muscle cramps, which strongly promote morphine use relapse. The morphine‐induced increases in neuroinflammation, brain oxidative stress, and alteration of glutamate levels in the hippocampus and nucleus accumbens have been associated with morphine dependence and a higher severity of withdrawal symptoms. Due to its rich content in potent anti‐inflammatory and antioxidant factors, secretome derived from human mesenchymal stem cells (hMSCs) is proposed as a preclinical therapeutic tool for the treatment of this complex neurological condition associated with neuroinflammation and brain oxidative stress.

**Methods:**

Two animal models of morphine dependence were used to evaluate the therapeutic efficacy of hMSC‐derived secretome in reducing morphine withdrawal signs. In the first model, rats were implanted subcutaneously with mini‐pumps which released morphine at a concentration of 10 mg/kg/day for seven days. Three days after pump implantation, animals were treated with a simultaneous intravenous and intranasal administration of hMSC‐derived secretome or vehicle, and withdrawal signs were precipitated on day seven by i.p. naloxone administration. In this model, brain alterations associated with withdrawal were also analyzed before withdrawal precipitation. In the second animal model, rats voluntarily consuming morphine for three weeks were intravenously and intranasally treated with hMSC‐derived secretome or vehicle, and withdrawal signs were induced by morphine deprivation.

**Results:**

In both animal models secretome administration induced a significant reduction of withdrawal signs, as shown by a reduction in a combined withdrawal score. Secretome administration also promoted a reduction in morphine‐induced neuroinflammation in the hippocampus and nucleus accumbens, while no changes were observed in extracellular glutamate levels in the nucleus accumbens.

**Conclusion:**

Data presented from two animal models of morphine dependence suggest that administration of secretome derived from hMSCs reduces the development of opioid withdrawal signs, which correlates with a reduction in neuroinflammation in the hippocampus and nucleus accumbens.

## Graphical abstract

Overall, findings indicate the generation of therapeutic benefits through the simultaneous intravenous and intranasal administration of secretome derived from preconditioned human mesenchymal stem cells, which helps mitigate morphine‐induced withdrawal syndrome in two relevant animal models of morphine dependence. These effects were associated with a reduction of morphine‐induced neuroinflammation in the hippocampus and nucleus accumbens.

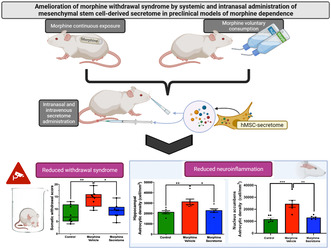

## 1 INTRODUCTION

The opioid dependence crisis has emerged as a significant global health concern,^1^ affecting millions of individuals, negatively impacting their physical, mental, and social well‐being. This situation has been exacerbated during the COVID‐19 pandemic, with the CDC's National Center for Health Statistics reporting an increase of nearly 15% in opioid overdose deaths in the United States from 2020 to 2021,^2^ with opioids responsible for almost 70% of all drug overdose deaths.^3^, ^4^ In Europe, the abuse of prescription opioids has also increased in the last two decades,^5^ with Scotland representing the country with the highest rates of opioid‐related harms.^5^, ^6^ Meanwhile, in South America, Chile is the country with the highest prevalence of drug use among adolescents, with opioid‐dependence incidence doubling in recent years.^7^

Morphine, a potent opiate analgesic, is frequently used for severe pain management but also has a high potential for abuse and dependence.^8^ One of the significant challenges faced by individuals attempting to discontinue morphine use is the withdrawal syndrome, a set of severe physiological and psychological symptoms experienced upon cessation or reduction of drug consumption.^9^ Withdrawal symptoms can vary in intensity depending on the duration and type of opioid abuse, but also by individual factors,^10^, ^11^ often leading to significant distress and discomfort. Consequently, a significant number of opioid‐dependent individuals return to opioid abuse to alleviate their symptoms,^12^ perpetuating the cycle of addiction and dependence.^13^

The main cause of withdrawal is related to the brain adaptation occurring while the individual is subjected to a constant opioid stimulus and subsequent μ‐opioid receptor activation,^14^ which is the main target of most commonly abused opioids, including morphine.^15^ Specifically, μ‐opioid receptor activation results in reduction of cAMP levels and increase in potassium channels activation.^14^ These effects reduce neuron excitability and affect brain areas with abundant μ‐opioid receptor expression, including areas related to affective responses like the hippocampus and the nucleus accumbens.^16^, ^17^ It is proposed that the brain responds to this increased opioid activation by increasing its excitatory tone, including increased glutamatergic activity.^14^, ^18^

Recent evidence suggests that the deregulated tone in glutamatergic activity is increased by glial immune activation. Opioids, including morphine, have been associated with an increase in both systemic and brain oxidative stress and inflammation.^19^, ^20^, ^21^, ^22^ Morphine can promote glial activation, and the subsequent increase in oxidative stress and inflammatory markers, by the direct activation of μ‐opioid receptors.^23^, ^24^ In addition, morphine activates a sensor of non‐endogenous molecules, the toll‐like receptor 4 (TLR4), promoting a pro‐inflammatory signaling cascade and upregulation of pro‐inflammatory cytokines.^25^ The resulting neuroinflammation has been shown to be persistent, promoting the increase of brain oxidative stress.^23^, ^26^, ^27^ An increase in neuroinflammation can alter behavior by modulating glutamatergic neurotransmission, first by upregulation of oxidant enzymes, and then by inactivation and downregulation of main glutamate transporters, like glutamate transporter‐1 (GLT‐1), in charge of glutamate uptake, and possibly the glutamate interchanger system X_c_^−^ and its catalytic subunit xCT, that promotes the release of extrasynaptic glutamate.^27^, ^28^, ^29^, ^30^ A reduction of these transporters levels and activity have been correlated to increased spillover of glutamate in the nucleus accumbens, increasing the activation of extrasynaptic glutamate receptors, in a model of heroin self‐administration and withdrawal.^31^ Consequently, an increase in GLT‐1 levels in the hippocampus and nucleus accumbens is correlated with reduced morphine and heroin relapse and withdrawal.^26^, ^31^, ^32^

Conventional treatments for opioid withdrawal syndrome include opioid maintenance therapy, in which long‐acting opioids like methadone are prescribed to replace short‐acting abused opioids like morphine or fentanyl. However, this therapy has limited efficacy since patients often return to taking the abused opioid when experiencing abstinence, and it does not address the brain adaptations that trigger withdrawal symptoms and promote relapse.^19^, ^33^, ^34^ Thus, there is an urgent need to explore novel therapeutic strategies to alleviate withdrawal symptoms, and handling opioid abstinence.

One promising approach for treating complex neurological diseases associated with neuroinflammation and brain oxidative stress is the use of secretome derived from human mesenchymal stem cells (hMSCs).^26^, ^35^, ^36^, ^37^, ^38^, ^39^ Secretome derived from hMSCs is a complex mixture of bioactive molecules, containing proteins, lipids, and regulatory microRNAs (miRNAs), with potent anti‐inflammatory, immunomodulatory, and regenerative properties, that can be easily obtained by the in vitro culture of these cells.^26^, ^40^ The secretome derived from MSCs has been tested at the preclinical level in different animal models of brain alterations associated with neuroinflammation and oxidative stress, including perinatal asphyxia,^41^ traumatic brain injury,^42^ lateral sclerosis,^43^ and alcohol or nicotine dependence.^39^ In all cases, secretome administration induced strong therapeutic effects without causing adverse reactions. Given the prominent role of oxidative stress and neuroinflammation in opioid withdrawal, the hMSCs secretome may offer a valuable therapeutic avenue for the management of morphine withdrawal syndrome.

In this preclinical study, we aimed at evaluating the therapeutic effects of the simultaneous intravenous and intranasal administration of hMSC‐derived secretome on morphine withdrawal, using two animal models of morphine dependence. This dual‐route approach for secretome administration was designed to maximize the therapeutic impact against opioid withdrawal, capitalizing on the unique benefits of each route. The intranasal method provides a non‐invasive delivery directly to the brain, leveraging the olfactory and trigeminal pathways to bypass the blood–brain barrier. In contrast, the intravenous route ensures a systemic distribution of the therapy, effectively addressing the widespread symptoms of opioid withdrawal. This combined approach, thus, targets both the central nervous system and systemic manifestations of opioid withdrawal.

In the first animal model, rats were implanted subcutaneously with osmotic mini‐pumps (Alzet) that infused morphine at a constant concentration of 10 mg/kg/day for seven days, and a withdrawal syndrome was precipitated by administration of the μ‐opioid antagonist naloxone three days after secretome administration. In this model, in an additional group of animals, we also measured oxidative stress and neuroinflammatory markers in the hippocampus and nucleus accumbens, and glutamate extracellular levels in the nucleus accumbens, to assess the impact of hMSC secretome before the withdrawal trigger. In the second animal model, rats voluntarily consumed morphine for 21 days, reaching an oral morphine consumption of 17 mg/kg/day. In this model, the withdrawal syndrome was spontaneously induced by morphine removal imposed immediately after secretome administration and evaluated 48 h later.

We hypothesized that secretome administration will decrease withdrawal behaviours and signs, evaluated in both preclinical models of morphine dependence, in addition to a reduction in brain oxidative stress and neuroinflammation, as well as normalization of glutamate levels.

## 2 MATERIALS AND METHODS

### 2.1 Animals

#### 2.1.1 Animal model 1. Subcutaneous morphine administration and withdrawal syndrome precipitation by naloxone

Eight‐week‐old female Wistar rats, weighing 180 to 220 g, were housed individually under controlled environmental conditions, including a 12‐h light/dark cycle and at a constant room temperature. They were under unrestricted access to rodent food and water, facilitating their natural behavioural patterns and minimizing extraneous stress.

#### 2.1.2 Animal model 2. Oral voluntary morphine consumption and spontaneous withdrawal syndrome precipitation by morphine deprivation

Just‐weaned three‐week‐old female Wistar rats weighing 50–65 g were single‐housed under a constant temperature, with a 12‐h light/dark cycle, and unrestricted access to standard rat chow and one bottle containing 0.15 mg/mL quinine hydrochloride (Sigma‐Aldrich) as the only water source to induce an adaptation to bitterness, facilitating the subsequent oral voluntary morphine consumption as previously described.^44^

Both animal models were conducted with female rats, as oral morphine consumption or its self‐administration is higher in females than in male rats.^45^, ^46^, ^47^ All animal protocols were approved by the Committee for Experiments with Laboratory Animals at the Universidad del Desarrollo (DCIM‐2021/02). The brain microdialysis assays were approved by the Animal Care Committee from the University of Valencia. The studies were conducted in accordance with Spanish laws (RD 53/2013) and the European Directive (EC 2010/63).

### 2.2 Morphine administration and evaluation of withdrawal syndrome

#### 2.2.1 Animal model 1: Subcutaneous morphine administration and withdrawal syndrome precipitation by naloxone

In this model, morphine dependence was developed in Wistar rats by implanting osmotic mini‐pumps subcutaneously (2ML2 ALZET®). These mini‐pumps were loaded with morphine hydrochloride (Sanderson Laboratory) to deliver a dose of 10 mg/kg/day, facilitating a steady and continuous release of the drug (5 μL/h) over seven days, following the manufacturer's instructions. Controls animals were implanted subcutaneously with osmotic mini‐pumps releasing a saline solution. This model simulates a state of morphine dependence in rats, mirroring the constant exposure to the drug in human scenarios.^48^

Three days post‐pump implantation, the morphine‐treated animals were randomly divided into two groups. One group received simultaneously an intranasal (25 μg of protein) and intravenous (25 μg of protein) dose of secretome derived from 1 × 10^6^ preconditioned human MSCs (*n* = 8), while the other group received the vehicle (*n* = 8). Both doses were administered in a volume of 160 μL. Animals of the control group (saline‐treated) received intranasal and intravenous administration of the vehicle. The dual‐route administration aims to simultaneously target the central and peripheral alterations induced by morphine dependence.

After seven days of continuous morphine exposure, rats were intraperitoneally injected with 5 mg/kg of the μ‐opioid receptor antagonist naloxone (Sigma‐Aldrich) dissolved in 0.9% saline. Naloxone administration triggered the sudden onset of a severe withdrawal syndrome,^49^ allowing for a controlled observation and analysis of the somatic signs associated with opioid withdrawal.

Immediately after the naloxone injection, each animal was placed in a glass beaker (300 mm in height and 180 mm in diameter) and monitored for a range of withdrawal symptoms for 30 min. These symptoms included weight loss, percentage of the area covered by feces, and somatic signs including jumps, ventral/dorsal flexes, stretching, chewing, burrowing, wet dog shakes, and forepaw tremors. In addition, vocalizations were recorded when the animal was held at the end of the induced withdrawal syndrome. The behavioral monitoring was conducted by two investigators who were blinded to the treatment conditions. The timeline of the experiments is depicted in Figure S1A.

#### 2.2.2 Animal model 2: Oral voluntary morphine consumption and spontaneous withdrawal syndrome precipitation by morphine abstinence

In this model, Wistar rats voluntarily ingested morphine over a three‐week period, leading to a state of morphine dependence as previously described,^44^ resembling the clinical situation of chronic morphine‐dependent patients. Since taste preference is a learned behavior, just‐weaned rats were trained to accept a bitter taste by adding the bitterant quinine hydrochloride (0.15 mg/mL) (Sigma‐Aldrich) to their drinking water for seven days, as previously reported.^44^ Following this forced exposure to quinine hydrochloride as the only fluid source, the animals transitioned to a two‐week regimen where they were given a choice between two bottles. One bottle contained quinine hydrochloride (0.15 mg/mL), and the other contained a solution of morphine sulfate (Oramoph, Molteni Farmaceutics; 0.15 mg/mL), both dissolved in tap water and with equivalent bitterness levels. After this period, the quinine bottle was removed, leaving the animals with a choice between tap water and morphine sulfate (0.15 mg/mL) for an additional week. A control group of animals had only access to water. To prevent side preference, the positions of the bottles were alternated daily. Morphine intake and preference, overall fluid consumption, and body weight were recorded daily. Unlike Model 1, where the withdrawal was precipitated by an μ‐opioid receptor antagonist, in this model, a withdrawal syndrome was induced by discontinuation of morphine access after three weeks of morphine exposure. On the same day, rats were administered simultaneously with intranasal and intravenous doses (same doses as Model 1) of either secretome or saline, depending upon the experimental group (Figure S1B). This allowed us to evaluate the spontaneous manifestation of somatic signs of morphine withdrawal, which were recorded for 30 min 48 h later. This approach simulates the natural course of withdrawal that would occur in an individual dependent on morphine who suddenly discontinues drug use, allowing us to test the effect of secretome administration during this stage.

In both animal models, a deprivation score was calculated based on the frequency of somatic withdrawal signs. This score was derived from a graduated scoring system for each parameter observed during a 30‐min evaluation period as previously reported.^50^, ^51^ The scale weighed signs considering their frequency (graded signs) or valued their presence if minimum events were observed (checked signs). Graded signs include climbing (score 1 if frequency between 1 and 25, score 2 if between 25 and 50, score 3 if >50), jumping (score 1 if frequency between 1 and 5, score 2 if between 5 and 10, score 3 if >10), chewing (score 1 if frequency between 1 and 5, score 2 if between 5 and 10, score 3 if >10), and abdominal constrictions (score 1, every 2 events). Checked signs include wet‐dog shakes (score 2 if >2), forepaw tremors (score 2 if >2), irritability (score 3 if observed), and diarrhea (score 3 if observed).

### 2.3 Isolation, expansion, and characterization of human adipose tissue‐derived MSCs

Human MSCs were isolated from fresh subcutaneous adipose tissue samples (abdominal region) obtained with written informed consent from healthy donors undergoing cosmetic liposuction at Clínica Alemana in Santiago, Chile. The donors were females aged between 22 and 56 years old, with a body mass index (BMI) of 25 ± 1 (Mean ± SEM). MSCs for a single donor were used in the present studies. The entire process was conducted under protocols approved by the Faculty of Medicine Ethics Committee at Clínica Alemana‐Universidad del Desarrollo, Santiago, Chile. hMSCs were isolated from the adipose tissue as previously described.^37^, ^52^ After two subcultures, the cells were characterized based on the criteria proposed by the International Society for Cellular Therapy,^53^ evaluating their phenotypic profile by surface marker expression and adipogenic, osteogenic and chondrogenic differentiation capabilities, thereby confirming the cells' identity as hMSCs.^37^, ^52^ One of the main advantages of using MSCs for therapeutic purposes is the low‐immunogenicity of the cells and their secreted molecules.^54^ This property allows for preclinical testing using xenogenic approaches, such as transplanting human MSCs or their secretome into animal models, enabling the evaluation of the efficacy of the secretome derived from human MSCs in animal models of various pathologies.^39^, ^41^, ^55^

### 2.4 hMSC preconditioning and secretome production

It has been reported that it is possible to modify the natural composition of MSC secretomes by subjecting these cells to an in vitro preconditioning stimulus. This leads to the secretion of an appropriate combination and ratio of bioactive molecules specific for a determined pathology.^56^ In this regard, our group and others have reported that the in vitro preconditioning of MSCs with pro‐inflammatory cytokines induces the production of high levels of anti‐inflammatory, antioxidant, and neuroprotective factors,^37^, ^56^ generating a biodug with enhanced therapeutic potential for the treatment of neurological conditions associated with increased neuroinflammation and oxidative stress. Preconditioning was achieved by incubating hMSCs at the third passage and 75% confluency in minimum essential medium (α‐MEM, Gibco) supplemented with 10% fetal bovine serum (FBS, HyClone) plus 10 ng/mL TNF‐α (R&D System) and 15 ng/mL IFN‐γ (R&D System) for 40 h.^39^, ^52^ After preconditioning, the hMSCs were exhaustively washed to remove the pro‐inflammatory cytokines and were cultured for an additional 48 h in α‐MEM, without FBS and phenol red as previously described.^37^ The culture medium (secretome) was then collected and centrifuged at 400 *g* for 10 min to remove any detached cells. The supernatant was subjected to a second centrifugation at 5000 *g* for 10 min to eliminate any residual cell debris. The secretome was then filtered using 0.22 μm filters and concentrated to fifty times its original volume using 3 kDa cutoff filters (Millipore). The protein concentration was determined using BCA protein assay kit (Thermo Scientific), and aliquots of the secretome were stored at −80°C pending further applications.

### 2.5 Non‐invasive administration of secretome derived from preconditioned hMSCs

Before secretome administration, rats were anaesthetized by an intramuscular administration of ketamine (60 mg/kg) and acepromazine (4 mg/kg),^57^ and then positioned in the supine position. A total of 160 μL of the secretome, containing 25 μg of proteins derived from 1 × 10^6^ preconditioned hMSCs, was administered intranasally. This was done by 20 μL droplets, alternately administered into each nostril with a pipette tip over a period of 40 min (four times into each nostril).^39^ Control animals received the same volume of saline solution. In addition to the intranasal route, the secretome was also administrated intravenously. This was performed immediately after intranasal delivery, with animals receiving a tail vein injection of 160 μL of secretome containing 25 μg of proteins derived from 1 × 10^6^ preconditioned hMSCs. The control group received an equivalent volume of saline solution.^26^

### 2.6 Assessment of morphine‐induced neuroinflammation

To evaluate neuroinflammation, we quantified the density of astrocytes and microglial cells in the hippocampus and the nucleus accumbens of rats, as previously described.^37^, ^58^ Rats were anesthetized by inhalation of 4% sevoflurane vapors (Baxter), and then intracardially perfused with 0.1 M PBS (pH 7.4) before euthanasia for brain sample collection.

Astrocyte and microglial density were evaluated by double‐labeling immunofluorescence against the astrocyte marker glial fibrillary acidic protein (GFAP), and the microglial marker ionized‐calcium‐binding adaptor molecule 1 (Iba‐1) in coronal 30 μm thick cryo‐sections of the hippocampus and nucleus accumbens, following previously reported methods.^59^ In brief, the coronal sections were washed with 0.1 M PBS and blocked for 1 h with a blocking solution (0.3% Triton X‐100, 0.1% BSA, and 10% normal goat serum in PBS). The sections were then incubated overnight at 4°C with a primary rabbit monoclonal anti‐IBA‐1 antibody (cat#019‐19741, Wako) at a dilution of 1:500 and with a primary mouse monoclonal anti‐GFAP antibody (cat#G3893, Sigma‐Aldrich) at a dilution of 1:500 in the blocking solution. After this incubation, the sections were rinsed with PBS containing 0.3% Triton X‐100, thereafter incubated for 2 h at room temperature in the dark, with a goat anti‐rabbit secondary antibody (Alexa Fluor 594, Thermo Fisher Scientific) at a dilution of 1:500 and a goat anti‐mouse secondary antibody (Alexa Fluor 488, Thermo Fisher Scientific) at a dilution of 1:500 in the blocking solution and counterstained with 4,6 diamino‐2‐phenylindol (DAPI, Thermo Fisher Scientific, 0.02 M; 0.0125 mg/mL) for nuclear labeling. Microphotographs were taken from the *Stratum Radiatum* of the hippocampus and the nucleus accumbens (NAc) using a confocal microscope (Olympus FV10i). The area analyzed for each stack measured 0.04 mm^2^, with the thickness (*Z* axis) being recorded for each case. The density of GFAP‐positive astrocytes and Iba‐1‐positive microglial cells was determined using the FIJI image analysis software as previously reported.^58^, ^59^

### 2.7 Assessment of morphine‐induced oxidative stress

To evaluate oxidative stress in the hippocampus of rats subjected to morphine exposure, with or without hMSC‐derived secretome administrations and under control conditions (no morphine), we measured the ratio of oxidized glutathione normalized by total protein (GSSG/protein) and the level of a lipid peroxidation marker (malondialdehyde, MDA, levels). Both measurements serve as indicators of oxidative stress.^26^, ^58^, ^60^ To evaluate the GSSG/protein ratio, we homogenized the hippocampus using a potassium buffer solution supplemented with 5 mM EDTA (pH 7.4). This homogenate was centrifuged at 18,000 *g* for 20 min, after which an aliquot was removed from the supernatant for protein measurement, before adding trichloroacetic acid (Sigma‐Aldrich, T0699) for protein precipitation. Then, the homogenate underwent a second round of centrifugation at 18,000 *g* for 15 min. In total, 20 μL of the resultant supernatant was treated with 0.5 μL of 2‐vinylpyridine (Sigma‐Aldrich, 100‐69‐6) to chemically mask reduced glutathione (GSH),^61^ preventing the initial binding of the thiol group from GSH with sulfhydryl reagent DTNB (Sigma‐Aldrich), as this union exhibits absorbance at 412 nm. Any excess of 2‐vinylpyridine was neutralized using triethanolamine. To evaluate the amount of GSSG, we first incubated the sample with β‐NADPH (Sigma‐Aldrich, N1630) and 5,5′‐dithiobis‐(2‐nitrobenzoic acid) (DTNB). Then, GSSG in the sample was converted into GSH by the addition of glutathione reductase (Sigma‐Aldrich, G3664) and incubated at 37°C in a microplate reader (Multiskan Sky, Thermo). GSH levels were measured by absorbance at 412 nm and the GSSG concentration in the sample was obtained using a calibration curve. Protein levels were measured by the BCA method (Thermo).

Lipid peroxidation was evaluated by measuring the formation of MDA, a product of lipid peroxidation. The MDA levels were assessed using the Lipid Peroxidation assay kit (Sigma‐Aldrich), following a previously reported method^26^, ^60^ and expressed as nmol MDA/mg protein.

### 2.8 Surgical implantation of the microdialysis probe

Under isoflurane anesthesia, the rat was positioned in a stereotaxic apparatus (Stoelting). After disinfecting the skin, a small incision was made over the skull, and the site was treated with 3% lidocaine gel for analgesia. The Lambda and Bregma points on the skull were identified to determine the placement of the microdialysis probes. Using these reference points, bilateral vertical concentric‐style microdialysis probes were implanted into the NAc (anteroposterior: +1.5 mm, mediolateral: ±1.6 mm, dorsoventral: −8.0 mm from Bregma).^62^ Holding screws were strategically inserted at two random points near the NAc cranial placement for enhanced stability. The probes were secured with dental cement, and the incision was sutured. After the surgery, the rats were allowed to recover in their cages. A 2ML2 ALZET® systemic continuous infusion pump, loaded with morphine or vehicle as described above for Animal Model 1, was also implanted during this procedure.

### 2.9 Assessment of glutamate levels by no‐net‐flux microdialysis

To evaluate the effect of secretome administration on glutamate levels in the context of morphine withdrawal syndrome, rats were divided into control, morphine‐saline, and morphine‐secretome. As described above for Animal Model 1, the control group was implanted with a mini‐pump filled with saline, and the morphine‐saline and morphine‐secretome groups were implanted with mini‐pumps containing morphine to deliver a constant dose of morphine (10 mg/kg/day) over seven days. Three days after pump implantation, the morphine‐secretome group received intranasal and intravenous doses of hMSCs‐secretome, while the other two groups received saline. Seven days post‐implantation, the microdialysis process was started. Probes were perfused with artificial cerebrospinal fluid (aCSF) at a rate of 2.5 μL/min. After an initial stabilization period of at least 1 h, samples were collected every 20 min to establish baseline glutamate levels. This was followed by the no‐net‐flux technique, which was used to precisely determine the extracellular glutamate levels in the implantation site by infusing increasing concentrations of glutamate (0.5; 5; 10 μM), with samples collected for each concentration.^31^ The placement of the cannula was confirmed post‐operation using cresyl violet staining (Figure S2). Only animals with correct microdialysis cannula placements were included in the data analyses.

To quantify glutamate concentration in the collected microdialysis samples, we employed a High‐Performance Liquid Chromatography (HPLC) system equipped with a fluorescence detector (Perkin‐Elmer series 200 HPLC System and Perkin Elmer Series 200 Fluorescence Detector for HPLC) for the measurement of the fluorescent product of glutamate derivatization. The system was set up with an excitation wavelength of 350 nm and an emission wavelength of 452 nm, reported as optimal parameters for glutamate determination.^63^ The mobile phase A was composed of 0.1 M sodium acetate adjusted to pH 6.9 with acetic acid, 2.5% methanol, and 2.5% tetrahydrofuran. The mobile phase B was 100% methanol. Mobile phase 1 was isocratically eluted at 1.5 mL/min, thereafter the mobile phase A was connected and allowed to equilibrate for 1 h before the start of the runs. For derivatization, a cocktail was prepared containing 27 mg of 2‐ophtalaldehyde (OPA) (Sigma‐Aldrich), dissolved in 500 μL of absolute ethanol, 4.5 mL of borate buffer (pH 9.3), and 20 μL of β‐mercaptoethanol (Sigma‐Aldrich).^64^ The samples were mixed 1:1 with the derivatization cocktail and a 30 μL aliquot of the derivatized sample was injected into the HPLC system. The glutamate‐derivative was retained using a reverse‐phase HPLC column C18, 5 μm; 50 × 4.6 mm (Ascentis® Express). Three minutes post‐injection, a gradient was manually initiated by connecting a bottle with 65% mobile phase A and 35% mobile phase B. After 1 min, the mobile phase was switched to 10% A and 90% B, and after another minute, it was changed to 100% A. The run was completed after 15 min, allowing the pressure system to return to the baseline level. The glutamate concentration in each sample was determined by comparing the peak heights with an external standard curve. This method allowed for precise quantification of glutamate levels in the NAc, providing insights into the effects of morphine withdrawal and hMSCs‐derived secretome treatment on glutamatergic balance in this key brain region.

### 2.10 Determination of xCT and GLT‐1 glutamate transporter levels in nucleus accumbens

Finally, we measured the protein levels of glutamate transporters GLT‐1 and xCT in the NAc using Western blot. For this, proteins from NAc were extracted using T‐PER lysis buffer (Thermo‐Fisher) supplemented with a protease inhibitor. 25 μg of protein were loaded into each lane. The GLT‐1 protein was identified using a guinea pig anti‐GLT‐1 primary antibody (Cat AB1783, Millipore, 1:500 dilution) and an IRDye 800CW donkey anti‐guinea pig secondary antibody (Cat 925‐32411, LI‐COR, 1:10,000 dilution). For xCT detection, a rabbit anti‐xCT primary antibody (Cat AB175186, Abcam, 1:500 dilution) was used in conjunction with an IRDye 800CW donkey anti‐rabbit secondary antibody (Cat 926‐32213, LI‐COR, 1:10,000 dilution). β‐actin, detected using a mouse anti‐β‐actin primary antibody (Cat sc‐47778 Santa Cruz Biotechnology, 1:200 dilution) and an IRDye 800CW goat anti‐mouse secondary antibody (Cat 926‐32210, LI‐COR), served as the loading control. The reactive bands were captured with the Odyssey Imaging System (LI‐COR), and the intensities were quantified using Image Studio Lite 5.2 software.

### 2.11 Statistical analysis

All statistical evaluations were conducted using GraphPad Prism software (9.2.0 version). Data are presented as means ± SEM. The Shapiro–Wilk test was used to verify the normality of the distribution for all experimental data. Bartlett's test was employed to confirm the homogeneity of variances between groups, with a *p*‐value >0.05 indicating equal variances. Upon satisfying these assumptions of normality and homogeneity, either one‐way or two‐way analysis of variance (ANOVA) was utilized, depending on the experimental conditions. Following the ANOVA, a Tukey post‐hoc test was performed to examine the differences between experimental groups. A *p*‐value <0.05 was considered to indicate statistical significance. To facilitate understanding and interpretation, the complete statistical analyses are provided in the figure legends.

## 3 RESULTS

### 3.1 Effects of secretome administration on somatic signs of morphine dependence and withdrawal score in the naloxone precipitated withdrawal model

**FIGURE 1:**
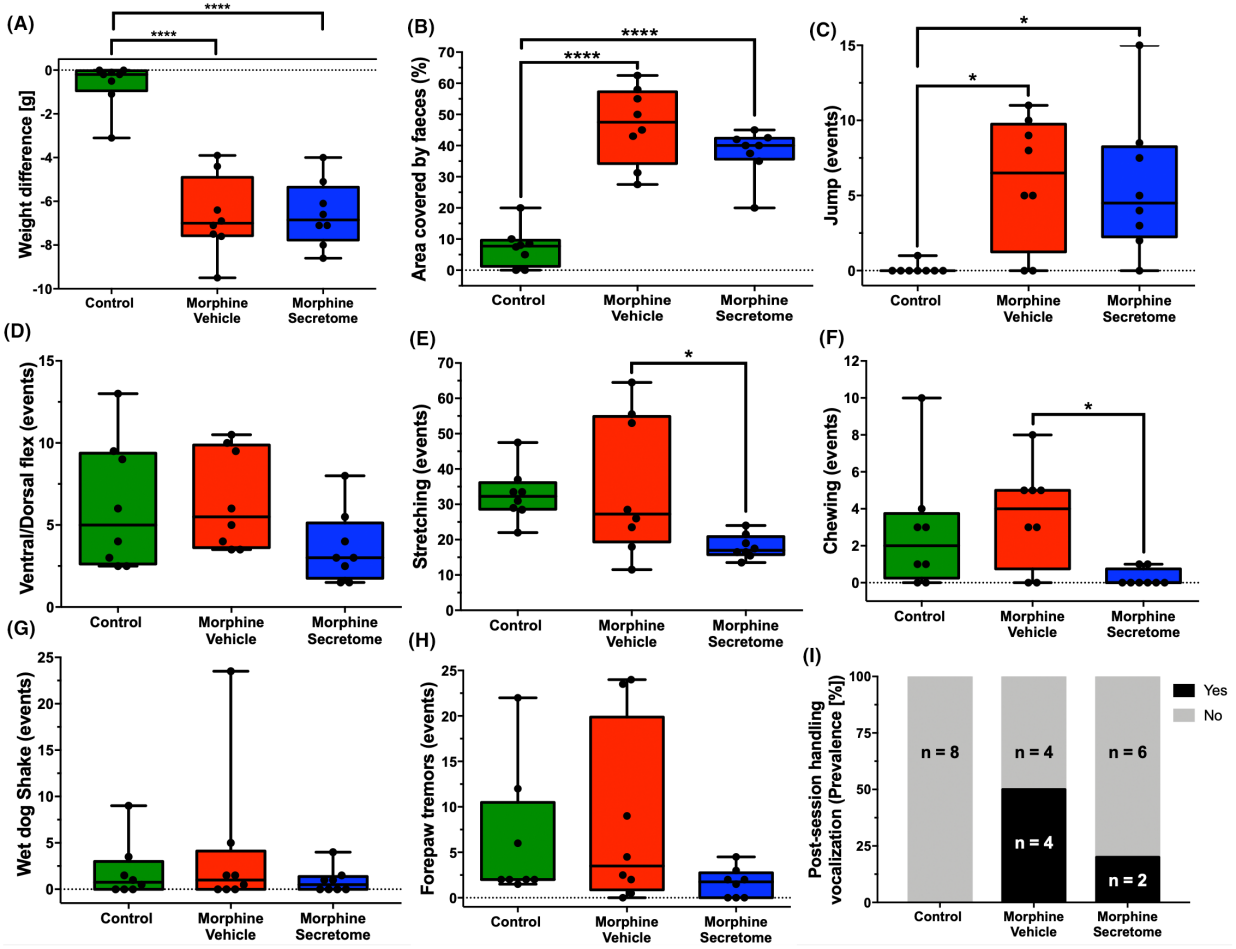
Effects of secretome administration on somatic signs of morphine dependence and withdrawal score in the naloxone‐precipitated withdrawal model. Eight‐week‐old female Wistar rats were implanted subcutaneously with osmotic pumps delivering morphine at a concentration of 10 mg/kg/day for seven days. On day three, animals received an intranasal and intravenous administration of MSC‐derived secretome (25 μg proteins derived from 1 × 10^6^ preconditioned hMSCs) or saline. Withdrawal syndrome was induced on day seven by the intraperitoneal administration of naloxone (5 mg/kg), after which behavior was recorded for 30 min to gauge the severity of signs. The following signs were evaluated: (A) weight differences, (B) area covered by feces, (C) jumps, (D) ventral/dorsal flexes, (E) stretching, (F) chewing, (G) wet dog shakes, (H) forepaw tremors events, and (I) post‐session handling vocalization prevalence. Control animals received saline solution from a continuous infusion pump; *n* = 8 per experimental condition. Data are presented as Min to Max. *p < 0.05; ****p < 0.0001; Tukey's multiple comparison test for normally distributed data and the Kruskal–Wallis test for non‐parametric data.

Previous work demonstrated that the intravenous and intranasal administration of secretome derived from preconditioned hMSCs significantly reduced oral morphine consumption in an animal model of voluntary morphine intake.^26^ Building on this observation, we sought to determine whether the hMSC‐derived secretome could also alleviate the withdrawal stage of opioid abuse. To this end, we comprehensively evaluated the somatic signs of withdrawal syndrome in morphine‐treated Wistar rats. Towards this aim, animals were implanted with subcutaneous osmotic mini‐pumps that delivered morphine (10 mg/kg/day) for seven days and treated both systemically and intranasally with MSC‐derived secretome or vehicle three days after pump implantation. On day seven, withdrawal syndrome was induced by the intraperitoneal administration of the μ‐opioid antagonist naloxone. The quantification of somatic withdrawal signs showed significant differences between the morphine‐vehicle and morphine‐secretome treated groups compared to the no‐morphine control group (Figure 1). These signs include weight difference before and after the induction of the withdrawal syndrome (*p* < 0.0001, One‐way ANOVA followed by Tukey's post‐hoc test) (Figure 1A), the area covered by feces (*p* < 0.0001, One‐way ANOVA followed by Tukey's post‐hoc test) (Figure 1B), and jump events (*p* < 0.05, One‐way ANOVA followed by Tukey's post‐hoc test) (Figure 1C). When comparing both groups administered with morphine, the morphine‐secretome group displayed a significant reduction in two somatic signs: stretching events (*p* < 0.05, One‐way ANOVA followed by Tukey's post‐hoc test) (Figure 1E) and chewing events (*p* < 0.05, One‐way ANOVA followed by Tukey's post‐hoc test) (Figure 1F) compared with the morphine‐vehicle group.

**FIGURE 2:**
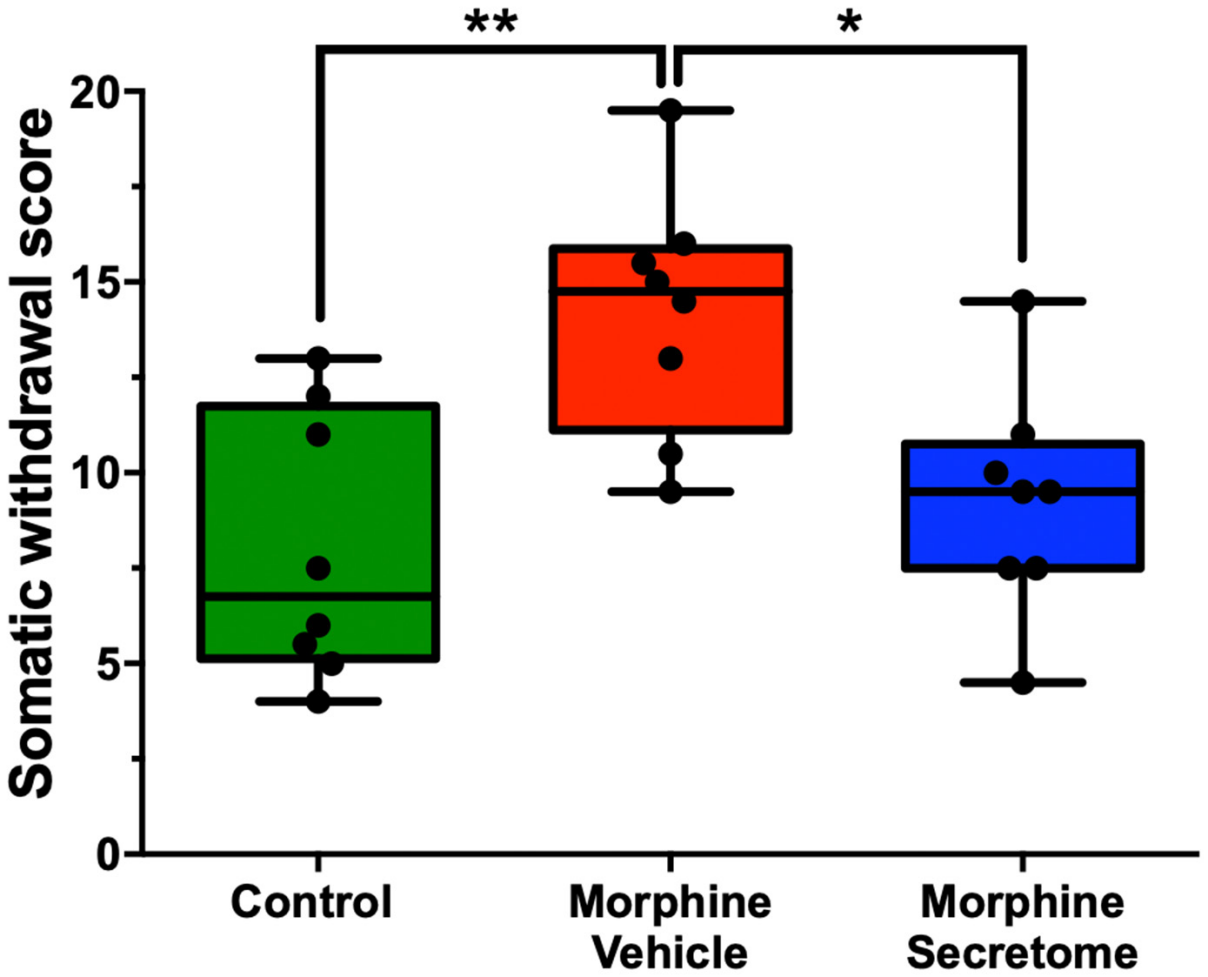
Effects of secretome administration on the combined withdrawal score in the naloxone precipitated withdrawal model. The somatic withdrawal is depicted using a scoring system based on previous studies.^50^, ^51^ Secretome treated rats showed a significant reduction in the somatic withdrawal score compared to morphine‐vehicle treated animals. Animals not exposed to morphine were considered controls. Data are presented as Min to Max; *n* = 8 for each experimental condition. *p < 0.05; **p < 0.01; Tukey's multiple comparison tests for normal distribution.

We also adapted a somatic withdrawal score based on the frequency of withdrawal symptoms (Table S1), as it has been previously reported that a withdrawal score provides a robust evaluation for the severity of withdrawal signs.^50^, ^51^ With this analytical methodology, we observed that rats exposed to morphine and treated with the vehicle displayed a significantly elevated somatic withdrawal score compared to the control rats (*p* < 0.01, One‐way ANOVA followed by Tukey's post‐hoc test) (Figure 2). However, this trend was notably altered following the administration of the hMSC‐derived secretome. Rats that were administered with secretome simultaneously intranasally and intravenously, exhibited a significant reduction in naloxone‐triggered withdrawal score compared to the group treated with the vehicle (*p* < 0.05, One‐way ANOVA followed by Tukey's post‐hoc test) (Figure 2).

### 3.2 Impact of secretome administration on astrocyte and microglial density in the hippocampus

**FIGURE 3:**
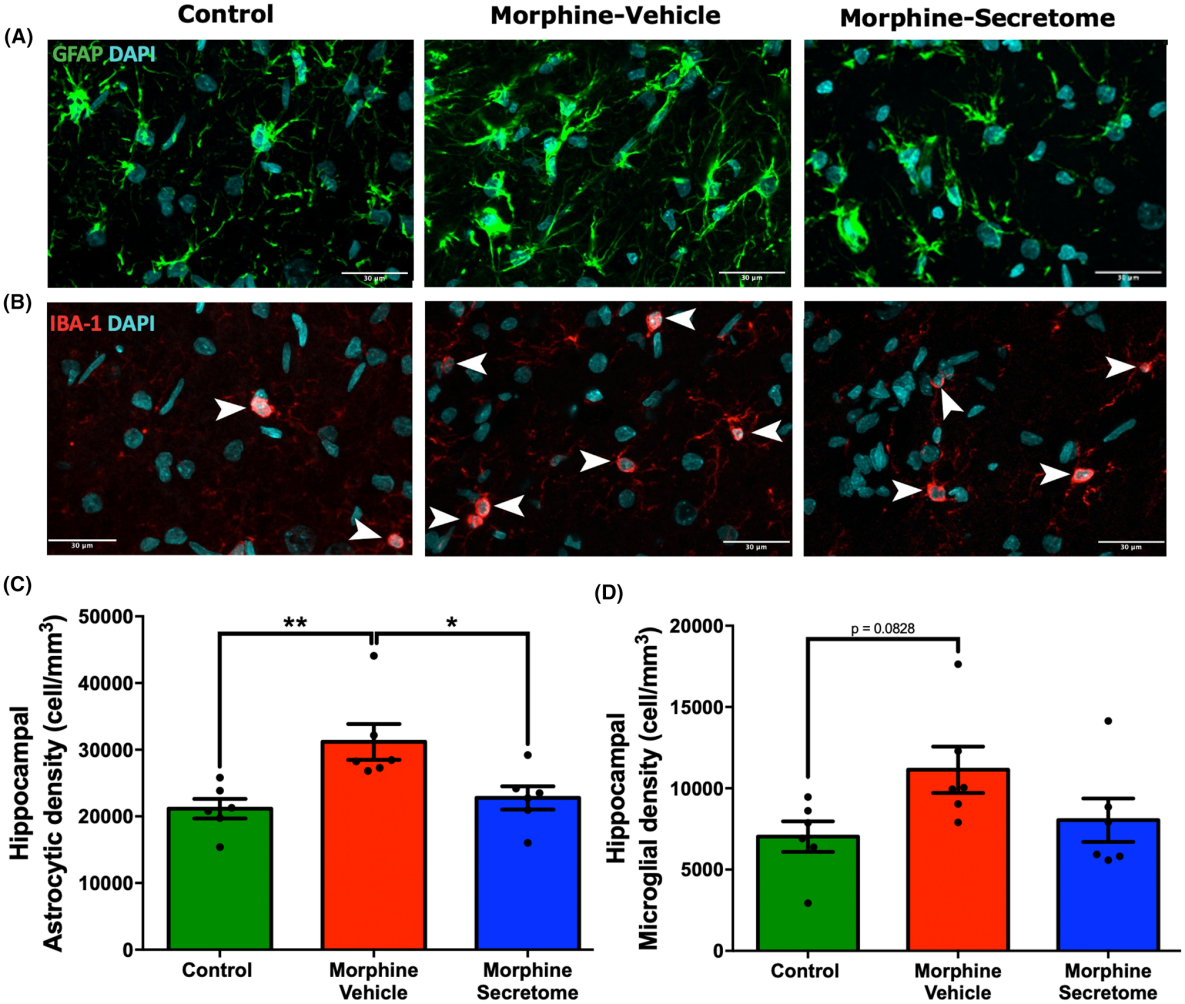
Effect of secretome administration on hippocampal astrocyte and microglial density. (A and B) Representative confocal microphotographs of GFAP immunoreactivity (depicted in green, top panel) of hippocampal astrocytes and Iba‐1 immunoreactivity (shown in red, indicated by white arrows, bottom panel) of hippocampal microglial cells. The nuclei were counterstained with DAPI (blue, nuclear marker); scale bar: 30 μm. (C) Quantification of astrocyte density. (D) Quantification of microglial density. Rats implanted with a morphine pump and treated with vehicle exhibited a significant increase in astrocyte density, but not in microglial density, compared to control rats. A single, simultaneous intranasal and intravenous administration of secretome three days after morphine‐pump implantation (morphine‐secretome) significantly reduced astrocyte density compared to that in morphine‐vehicle treated rats. Data are presented as mean ± SEM. *n* = 6 for each experimental condition. *p < 0.05, **p < 0.01 One‐way ANOVA followed by Tukey's post‐hoc test.

Considering the pivotal role of the hippocampus in associative memory networks, the encoding and consolidation of novel environmental information, and its documented contribution to the development of drug‐seeking memory behavior,^65^, ^66^ we evaluated neuroinflammation in this brain region in the morphine antagonist‐triggered withdrawal model prior to naloxone administration. This evaluation holds particular relevance in light of evidence indicating the occurrence of hippocampal neuroinflammation caused by naloxone administration in a morphine dependence rat model.^67^ As expected, rats that received morphine exhibited a significant increase in astrocyte density compared to control rats (*p* < 0.01, One‐way ANOVA followed by Tukey's post‐hoc test) (Figure 3A,C). A similar trend was observed for microglial density. However, this increase did not reach statistical significance (*p* = 0.0828, One‐way ANOVA followed by Tukey's post‐hoc test) (Figure 3B,D). By contrast, rats that received morphine but were treated with secretome showed a significant reduction in the increased astrocyte density in the hippocampus (*p* < 0.05, One‐way ANOVA followed by Tukey's post‐hoc test) (Figure 3A,C), while microglia density tended to decrease (Figure 3B,D).

### 3.3 Effects of secretome administration on hippocampal oxidative stress markers

**FIGURE 4:**
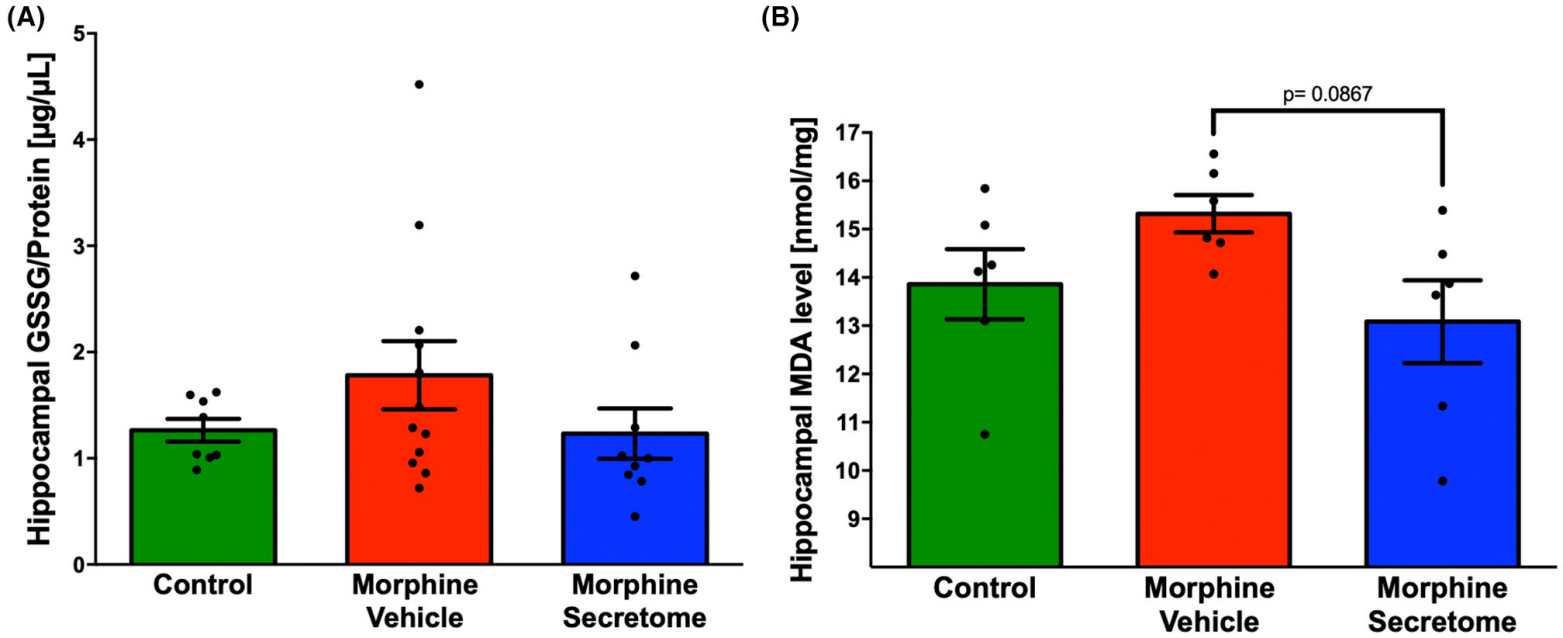
Evaluation of morphine administration and secretome administration on oxidative stress markers in the hippocampus. (A) GSSG/protein ratio in the hippocampus. (B) Malondialdehyde (MDA) levels in the hippocampus. Rats infused with morphine for seven days and treated with vehicle did not show a significant increase in GSSG/protein ratio or MDA levels compared to no‐morphine control rats. A single, simultaneous intranasal and intravenous administration of secretome did not alter GSSG/protein ratio or MDA levels. Data are presented as mean ± SEM; *n* = 6 to 8 for each experimental condition. One‐way ANOVA followed by Tukey's post‐hoc test.

Given the substantial evidence of the presence of oxidative stress during chronic morphine consumption,^26^, ^68^ and the withdrawal stage,^22^, ^69^ we evaluated the presence of hippocampal oxidative stress in the morphine antagonist‐triggered withdrawal model prior to naloxone administration. However, the evaluation of oxidative stress markers, including GSSG levels and MDA levels, did not reveal significant changes during the seven‐day morphine exposure in the morphine‐vehicle group compared to the control group (Figure 4A,B). Similarly, secretome administration only trended to reduce hippocampal MDA levels (*p* = 0.08, One‐way ANOVA followed by Tukey's post‐hoc test) compared to animals treated with vehicle (Figure 4B), suggesting that seven days of morphine exposure is not enough to induce a significant increase in hippocampal oxidative stress.

### 3.4 Impact of secretome administration on astrocyte and microglial density in the nucleus accumbens

**FIGURE 5:**
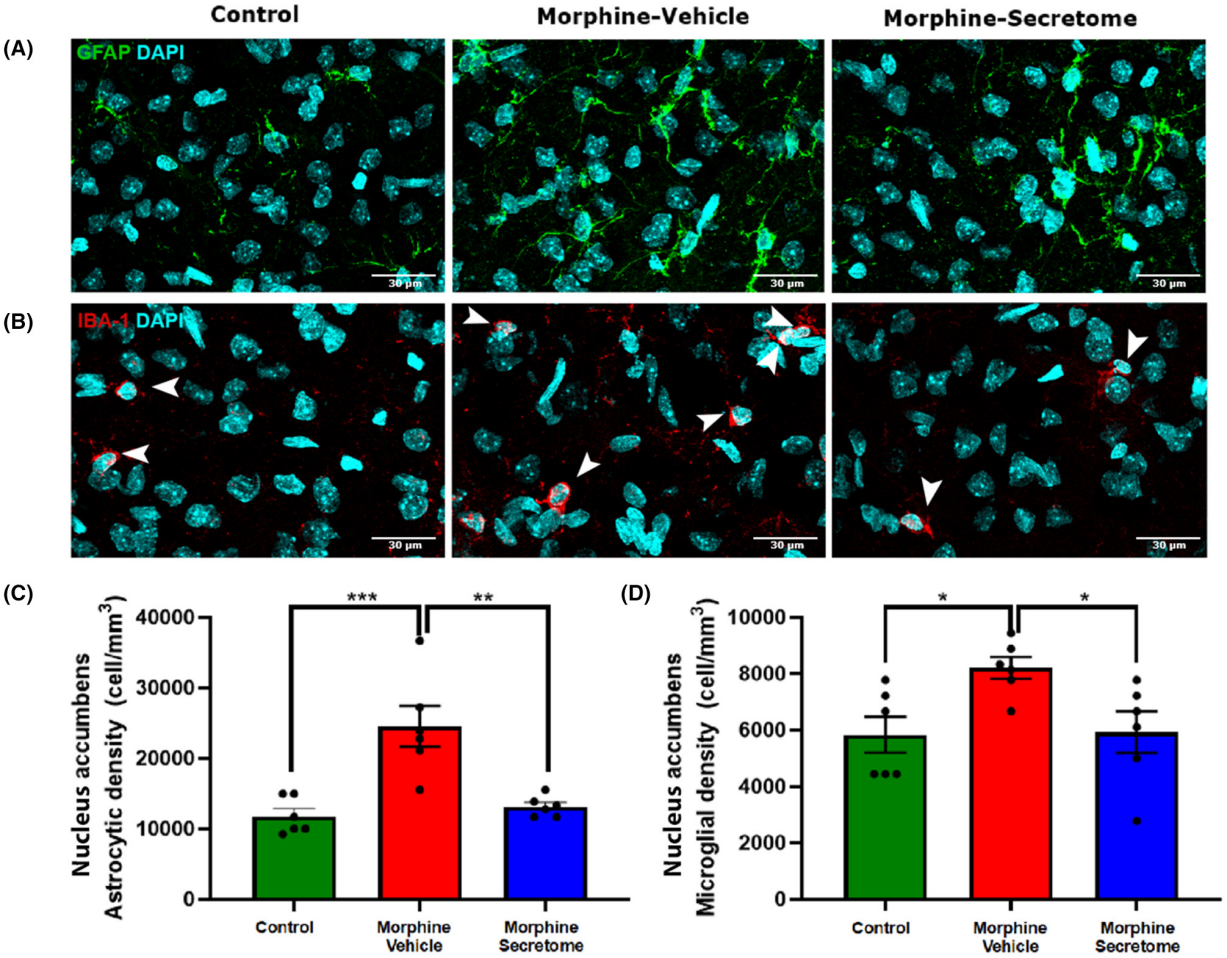
Effect of secretome administration on the morphine‐induced increases in astrocyte and microglial density in nucleus accumbens. (A and B) Representative confocal microphotographs of GFAP immunoreactivity (depicted in green, top panel) of hippocampal astrocytes and Iba‐1 immunoreactivity (shown in red, indicated by white arrows, bottom panel) of hippocampal microglial cells. The nuclei were counterstained with DAPI (blue, nuclear marker); scale bar: 30 μm. (C) Quantification of astrocyte density. (D) Quantification of microglial density. Rats implanted with a morphine pump and treated with a vehicle exhibited a significant increase in astrocyte density and in microglial density, compared to control rats. A single, simultaneous intranasal and intravenous administration of secretome three days after morphine‐pump implantation significantly reduced astrocyte and microglial density compared to vehicle treated rats. Data are presented as mean ± SEM. *n* = 6 for each experimental condition. *p < 0.05, **p < 0.01 One‐way ANOVA followed by Tukey's post‐hoc test.

We subsequently centred our focus on the nucleus accumbens inflammatory state. In this brain area, animals that received morphine showed a significant increase in astrocyte (Figure 5A,C) and microglial density (Figure 5B,D) compared to that in control rats (*p* < 0.01, One‐way ANOVA followed by Tukey's post‐hoc test). Importantly, rats that received morphine and were treated with secretome showed a significant reduction in the increased astrocyte (Figure 5A,C) and microglial density (Figure 5B,D) (*p* < 0.05, One‐way ANOVA followed by Tukey's post‐hoc test).

### 3.5 Effects of secretome administration on the modulation of nucleus accumbens glutamate levels and the expression of glutamate transporters GLT‐1 and xCT

**FIGURE 6:**
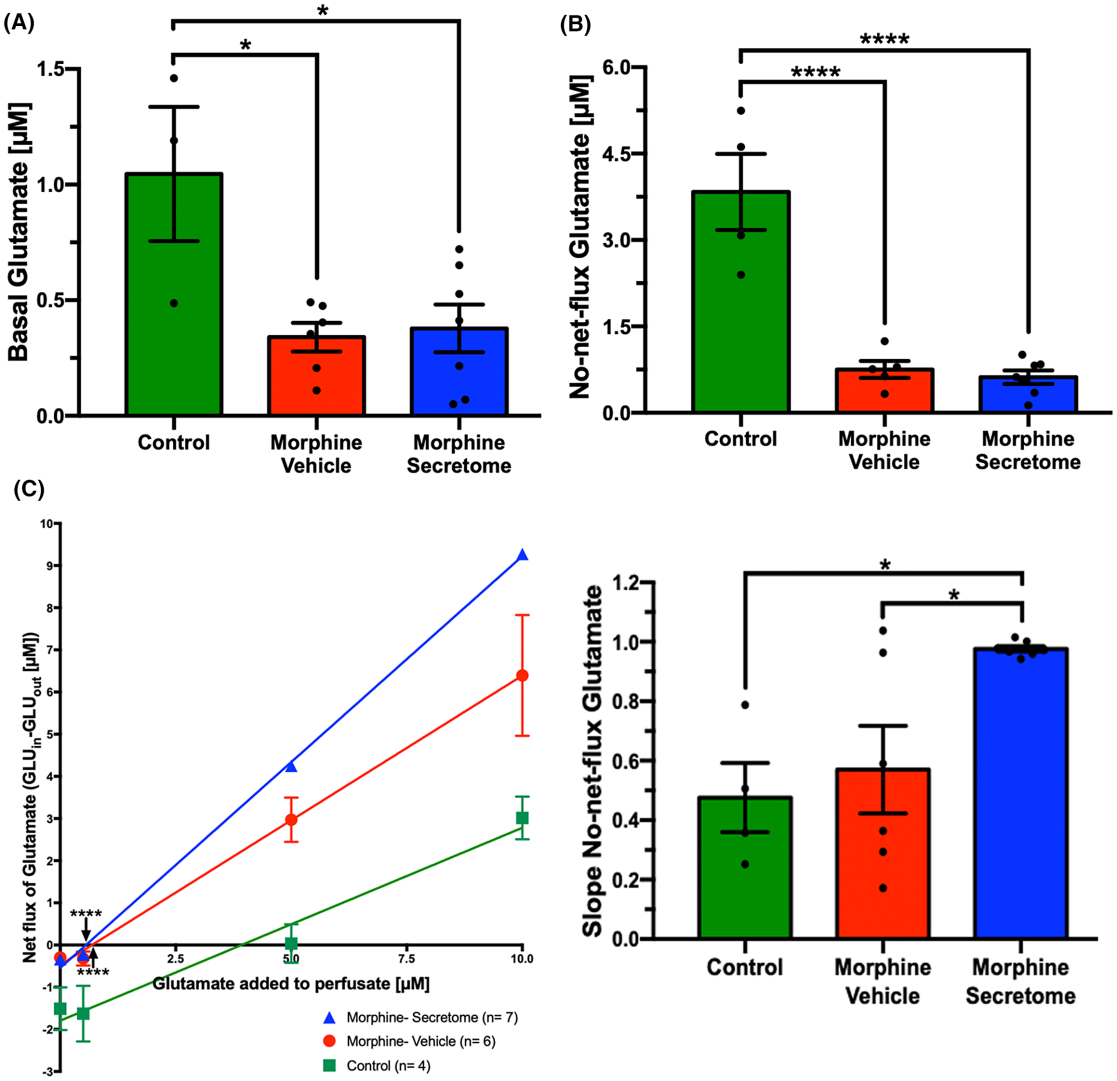
Evaluation of secretome administration effects on glutamate levels in nucleus accumbens. Eight‐week‐old female Wistar rats were implanted subcutaneously with osmotic pumps delivering morphine at a concentration 10 mg/kg/day. In the same procedure rats underwent microdialysis probe implantation in the NAc. Control animals received saline‐loaded pumps (*n* = 4), while morphine‐saline (*n* = 6) and morphine‐secretome (*n* = 7) groups received morphine‐loaded pumps (10 mg/kg/day). Three days post pump implantation, secretome or vehicle was administrated and seven days post pump implantation, no‐net‐flux microdialysis was used to determine NAc glutamate levels. Following a 1‐h stabilization period, samples were collected every 20 min for basal glutamate levels. Then, increasing concentrations of glutamate (0.5 μM, 5 μM, 10 μM) were perfused. (A) Quantification of basal glutamate concentrations average. (B) No‐net‐flux glutamate concentration. (C) No‐net‐flux plots (glutamate concentration set to enter the probe (GLU_in_)—glutamate concentration output (GLU_out_) v/s GLU_in_) and quantification of the slopes obtained from no‐net‐flow curves. Both experimental groups exposed to morphine significantly reduced the extracellular glutamate concentration, compared to the control group. Data are presented as mean ± SEM. *p < 0.05, ****p < 0.0001, One‐way ANOVA followed by Tukey's post‐hoc test.

Following the observation that secretome treatment promoted a reduction in NAc neuroinflammatory markers, we examined the effects of secretome administration on extracellular glutamate levels in this brain region. A one‐way ANOVA revealed a significant reduction versus no‐morphine controls in both the basal glutamate levels (Figure 6A) and in the no‐net‐flux glutamate levels (Figure 6B) in the NAc for both the morphine‐vehicle and morphine‐secretome treated animals (*p* < 0.0001, One‐way ANOVA followed by Tukey's post‐hoc test) compared to the control group, suggesting a reduced spillover of extracellular glutamate. No significant difference was observed among glutamate levels measured in morphine exposed groups treated with secretome or vehicle (*p* < 0.05, One‐way ANOVA followed by Tukey's post‐hoc test) (Figure 6A,B). However, a significant increase in the slope of the no‐net‐flux curve was observed in the morphine‐secretome group compared to the control groups (Figure 6C), which could indicate a modified glutamate transport dynamic.

**FIGURE 7:**
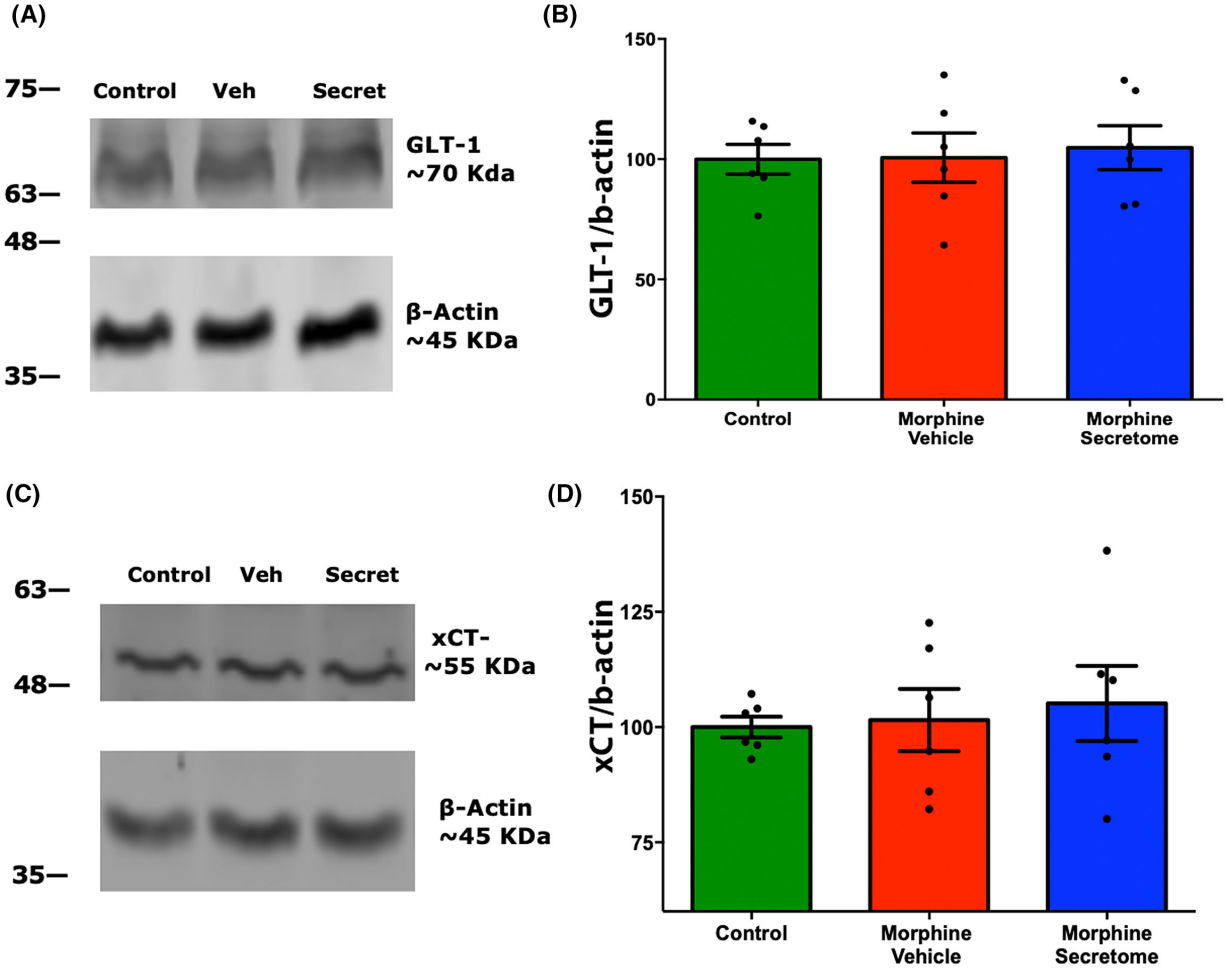
Evaluation of secretome effects on GLT‐1 and xCT glutamate transporter levels in nucleus accumbens. (A) Representative images of Western Blot for the glutamate transporter GLT‐1 in NAc. (B) Quantification of GLT‐1 to β‐actin levels in NAc. (C) Representative images of Western Blot for the glutamate transporter xCT in NAc. (D) Quantification of xCT to β‐actin levels in NAc. No significant differences were observed in the expression of GLT‐1 and the xCT‐system within the NAc across the three experimental groups (rats infused with morphine for seven days and treated with vehicle, rats infused with morphine for seven days and treated with secretome and no‐morphine control rats). Data are presented as mean ± SEM, *n* = 6 for each experimental group. One‐way ANOVA followed by Tukey's post‐hoc test.

The role of glial glutamate transporters in controlling glutamate uptake has been previously associated with the emergence of morphine dependence.^32^, ^70^, ^71^ However, we did not observe any significant changes either in the levels of the glutamate transporters GLT‐1 (Figure 7A,B) or the xCT system (Figure 7C,D) in NAc homogenates among all experimental groups.

### 3.6 Effects of secretome administration on somatic signs of morphine dependence and withdrawal score following deprivation after chronic oral voluntary morphine consumption

**FIGURE 8:**
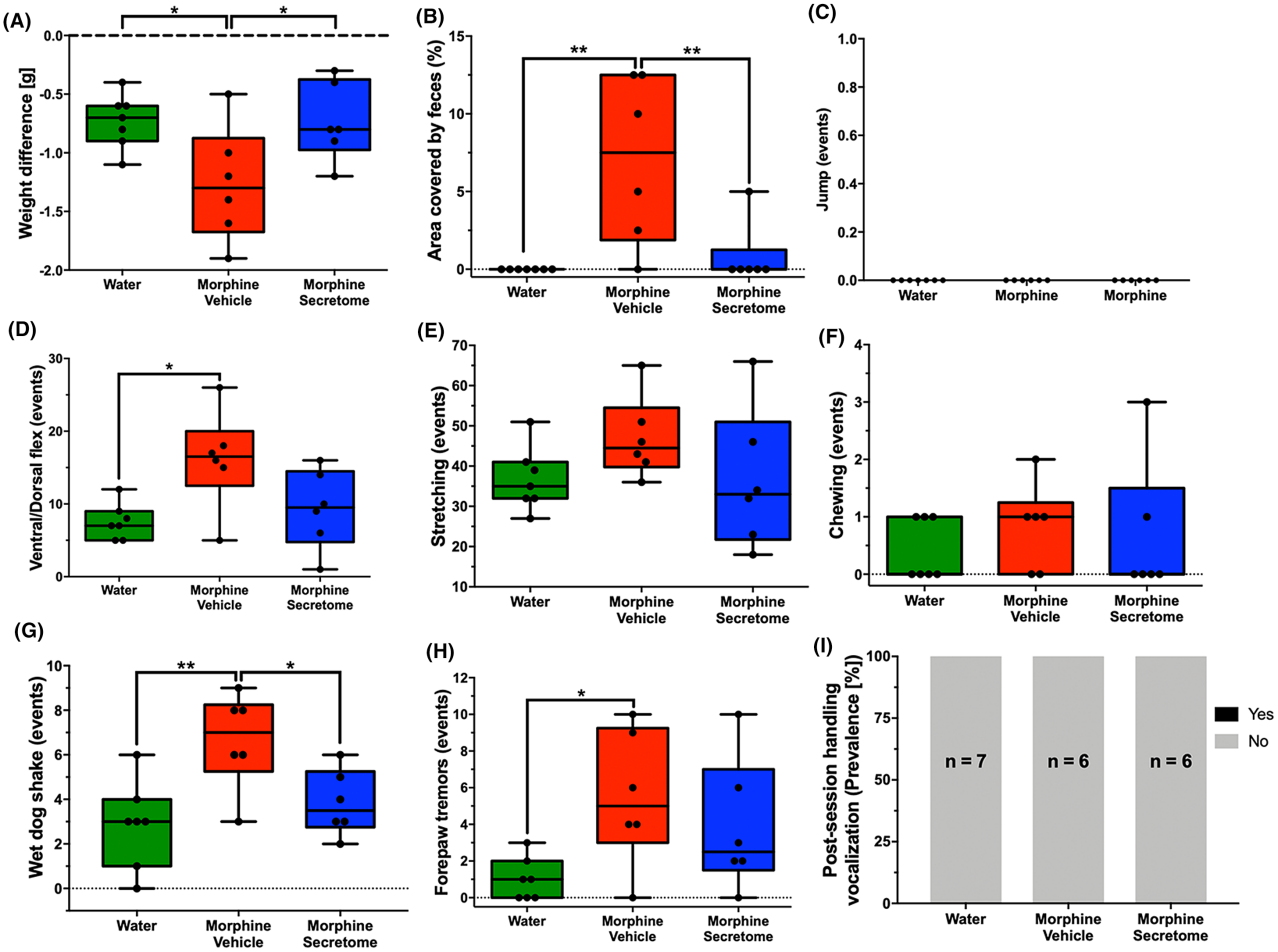
Effects of secretome administration on somatic signs of morphine dependence after morphine deprivation in an animal model of oral voluntary morphine consumption. Eight week‐old female Wistar rats voluntarily consumed morphine (~17 mg/kg/day) for three weeks. At such time the animals received a simultaneous intranasal and intravenous administration of MSC‐derived secretome (containing 25 μg proteins derived from 1 × 10^6^ hMSCs) or saline. On the same day, spontaneous withdrawal syndrome was induced by removing the morphine bottle. Somatic signs of withdrawal syndrome were evaluated 48 h post‐morphine removal during a 30‐min observation period. The assessed symptoms included (A) weight differences, (B) area covered by feces, (C) jumps, (D) ventral/dorsal flexes, (E) stretching, (F) chewing, (G) wet dog shakes, (H) forepaw tremors events and (I) post‐session handling vocalization prevalence. Control group animals had access only to water. Data are presented as min to max (*n* = 6–7 per experimental group). *p < 0.05; **p < 0.01; Tukey's multiple comparison tests for normal distribution and non‐parametric data evaluated using the Kruskal–Wallis test.

**FIGURE 9:**
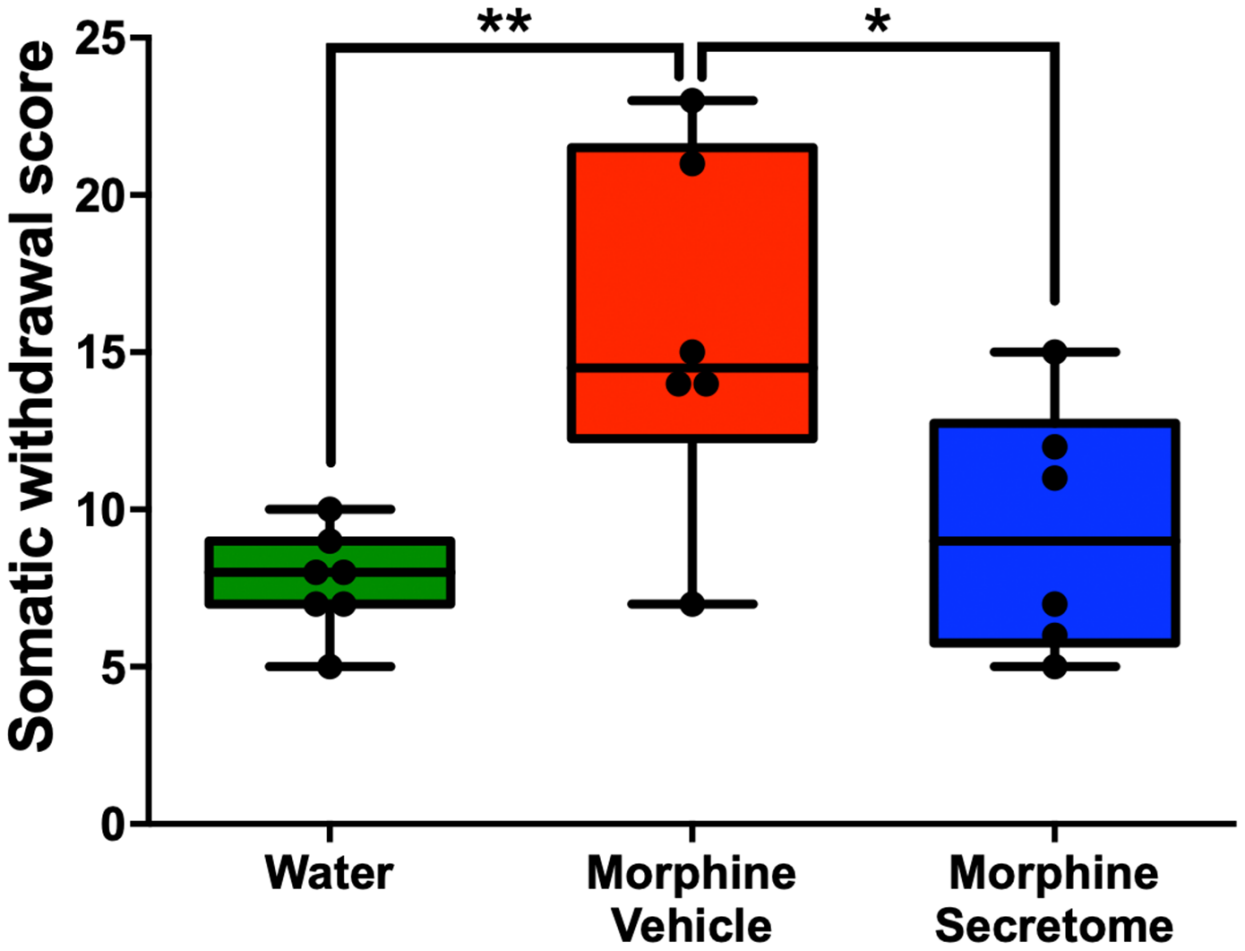
Effects of secretome administration on the combined withdrawal score after morphine deprivation in an animal model of oral voluntary morphine consumption. The somatic withdrawal is depicted using a scoring system based on previous studies.^50^, ^51^ Secretome treated rats showed a significant reduction in the somatic withdrawal score compared to vehicle‐treated animals. Control group animals had access only to water. Data are presented as min to max *n* = 6 in morphine and *n* = 7 in the control groups. *p < 0.05; **p < 0.01; Tukey's multiple comparison test for normal distribution.

Finally, we examined the effects of the intravenous and intranasal administration of secretome on the somatic signs of spontaneous withdrawal syndrome in an animal model of chronic voluntary oral morphine consumption, 48 h after morphine deprivation. This time point was selected based on clinical reports suggesting that the peak of withdrawal symptoms typically occurs between 36 and 72 h following the last opioid administration.^34^ Furthermore, another study in rats detected morphine levels below the quantitative limits but above background noise 48 h after subcutaneous morphine administration.^72^ At this time point, it is expected that the concentration of the opioid has diminished to less than 6.25% of the initial dose.^73^ For this, we used a recently reported animal model of opioid dependence in which young Wistar rats were initially acclimated to a bitter taste by exposing them to a bitter quinine solution as their only fluid source for seven days.^44^ After this period, rats were given a free choice between quinine (15 mg/mL) or morphine (15 mg/mL) solutions (two‐bottle choice) for two weeks. During this time, rats exhibited a high morphine consumption rate of 20.2 ± 2.1 mg/kg/day. Finally, rats were offered a choice between a morphine solution (15 mg/mL) and water for one additional week. This led to a voluntary morphine intake of 16.8 ± 2.2 mg/kg/day (Figure S3A), with a preference for the morphine solution over water exceeding 90% (Figure S3B). After three weeks of voluntary morphine intake, animals were both deprived of morphine and treated with an intravenous and intranasal administration of secretome or vehicle. Forty‐eight hours later, the withdrawal signs were evaluated for 30 min. We observed that upon withdrawal, morphine‐exposed rats treated with the vehicle showed a significant increase in different somatic signs compared to only water exposed animals, including weight difference (*p* < 0.05, One‐way ANOVA followed by Tukey's post‐hoc test) (Figure 8A), the area covered by feces (*p* < 0.001, One‐way ANOVA followed by Tukey's post‐hoc test) (Figure 8B), the number of ventral/dorsal flexions (*p* < 0.05, One‐way ANOVA followed by Tukey's post‐hoc test) (Figure 8D), the number of wet dog shakes (*p* < 0.001, One‐way ANOVA followed by Tukey's post‐hoc test) (Figure 8G), and the number of forepaw tremors (*p* < 0.05, One‐way ANOVA followed by Tukey's post‐hoc test) (Figure 8H). Contrasting the above, secretome‐treated animals showed a significant reduction in many of the morphine‐induced withdrawal signs, including weight difference (*p* < 0.05, One‐way ANOVA followed by Tukey's post‐hoc test) (Figure 8A), the area covered by feces (*p* < 0.001, One‐way ANOVA followed by Tukey's post‐hoc test) (Figure 8B) and the number of wet dog shakes (*p* < 0.05, One‐way ANOVA followed by Tukey's post‐hoc test) (Figure 8G) compared to vehicle treated animals. Importantly, the combined withdrawal score of morphine‐secretome treated animals was markedly lower than that for morphine‐vehicle treated animals (*p* < 0.05, One‐way ANOVA followed by Tukey's post‐hoc test) (Figure 9).

The combined above findings underscore the potential therapeutic role of the secretome in reducing symptoms of morphine withdrawal.

## 4 DISCUSSION

The present study examines the effects of simultaneous systemic and intranasal administration of secretome derived from preconditioned human mesenchymal stem cells on morphine‐induced withdrawal syndrome in rats. The findings encompass behavioral, cellular, and molecular aspects that have been previously described to be altered by chronic morphine administration.^44^ In both animal models tested, we observed that somatic withdrawal signs were heterogeneously distributed, indicating that some animals exhibit a significant increase in some of the analyzed parameters, while others show fewer alterations on these parameters. This pattern was noted across all three studied groups: control, morphine‐vehicle, and morphine‐secretome. This variability in behavior has been previously reported and is a primary reason for the literature to present a wide range of withdrawal parameters to be scored.^10^, ^74^ Consequently, we consolidated these behavioral alterations into a somatic withdrawal score, which was validated based on previous studies^50^, ^51^ and adapted considering the intensity and frequency of each parameter observed in our study. In both animal models, secretome administration markedly reduced the withdrawal score.

Previous reports have established that the administration of secretome derived from hMSCs can reduce chronic consumption and relapse of substances of abuse, including alcohol,^37^, ^39^ nicotine,^39^ and morphine.^26^ Our study pioneers in demonstrating that this biodrug can also alleviate the morphine withdrawal syndrome in two animal models of morphine dependence. This is a major finding, considering that the withdrawal syndrome often triggers the relapse into opioid consumption, as needed to avoid the somatic signs induced by opioid deprivation,^34^, ^74^, ^75^ thus impeding morphine discontinuation.^76^, ^77^, ^78^

Noteworthy, we observed this positive outcome following the simultaneous intravenous and intranasal administration of the hMSC‐derived secretome. In our previous studies on alcohol and nicotine addiction, we found that the intranasal administration of the hMSC‐derived secretome effectively reduced substance abuse.^39^ However, when evaluating the therapeutic effect of secretome on opioid dependence, we incorporated an additional administration route, following findings from other studies.^26^, ^79^ One of these studies showed that the intravenous administration of MSCs significantly reduced morphine‐induced inflammation in the spinal cord, a peripheral alteration associated with opioid tolerance.^79^ Moreover, a preclinical rat model of morphine exposure during six days showed an increase in plasma IL‐1β and IL‐6 levels, but intriguingly, the same cytokines did not increase at brain level.^80^ There are studies that have highlighted persistent systemic inflammation in drug users even after complete abstinence.^21^ This inflammation has been characterized by strong inflammatory responses during the early stage of abstinence, which then gradually alleviate along with the withdrawal time. However, even after 12 months of abstinence, immune dysfunction still exists and may persist for longer times in heroin and methamphetamine users.^21^ Therefore, in this report, we opted for a simultaneous intranasal and intravenous administration of the hMSC‐derived secretome. The strategy with a dual‐route administration aims to comprehensively address these complex central and peripheral changes induced by morphine dependence, providing a more effective treatment strategy. However, it's important to note that while our dual‐route administration strategy is based on previous research, direct evidence demonstrating its superior efficacy compared to a single‐route administration is still needed. The intranasal route has also recently been proposed for the preclinical evaluation of the effects of the competitive opioid receptor antagonist naltrexone in reducing opioid side effects. In this report, low‐dose naltrexone intranasal administration given 30 min before opioid administration reduced cognitive impairments and motor alterations induced by morphine administration in mice.^81^ Thus, suggesting that the intranasal route is an effective route for various therapeutics to reach the brain in the context of opioid use.

In the animal model of continuous morphine administration, secretome administration effectively mitigated the neuroinflammation that developed before the naloxone‐triggered withdrawal stage. This was evidenced by the full reversal of the morphine‐induced increase in astrocyte density within the hippocampus and astrocyte and microglial density in nucleus accumbens, regions crucially associated with memory consolidation^82^ and brain reward circuitry.^83^ Further research is needed to determine whether the increased astrocyte and microglial density observed after continuous subcutaneous morphine administration is also associated with an increase in astrocyte and microglial activation.

The observed impact of secretome administration on astrocyte and microglial density in morphine‐dependent rats underscores a potential mechanism through which the secretome may exerts its therapeutic effects. The increase in astrocyte and microglial density following morphine administration aligns with findings indicating that opioids can activate different pro‐inflammatory pathways.^25^, ^80^, ^84^ This activation could potentially occur via direct downstream signaling of the μ‐opioid receptor,^85^ as well as via the activation of the TLR‐4 signaling pathway, binding to the adaptor molecule MD‐2, within the central nervous system.^25^, ^86^ Recent studies have described the role of morphine to induce peripheral inflammation, specifically by inducing dysbiosis and amplifying bacterial translocation from the intestine to the liver and mesenteric lymph nodes.^87^, ^88^, ^89^ This cascade of events potentially triggers a systemic pro‐inflammatory state,^90^, ^91^ thereby underscoring a new mechanism of action in opioid dependence that merits further exploration. These findings highlight the complex interplay among opioid addiction, secretome treatment, and neuroinflammation, providing information for future studies to evaluate these mechanisms.

While there is compelling evidence suggesting that like many other drugs of abuse,^27^ morphine can induce an increase in oxidative stress^68^ in cultures of hippocampal neurons^92^ and in hippocampal tissue from rats exposed to morphine,^93^ we did not observe a corresponding increase in oxidative stress biomarkers in the hippocampal tissue, evaluated both by determination of GSSG and MDA levels after the seven‐day morphine exposure in animal model 1. The reasons for these differences are not clear but may be associated with several factors. Recent evidence suggests that the effects of morphine on oxidative stress may be dose‐ and time‐dependent.^94^, ^95^, ^96^ This is aligned with previous findings in an animal model of voluntary morphine intake, in which four weeks of morphine consumption induced a three‐fold increase in GSSG/GSH ratio and MDA levels in the hippocampus compared with rats drinking only water,^26^ increases that were fully normalized by hMSC‐derived secretome administration.^26^ Another possible explanation for the absence of increased oxidative stress in our animal model could be attributed to sex differences observed in response to opioid exposure.^97^, ^98^, ^99^ A significant proportion of the reports showing an increase in brain oxidative stress are based on studies conducted in male rats,^69^, ^100^, ^101^, ^102^, ^103^, ^104^ unlike the present studies in female rats, suggesting that sex of the animal might also be an important variable.^105^

Neuroinflammation and oxidative stress self‐potentiate each other,^106^ and both processes have been associated with a reduction of the levels and activity of the key glutamate transporters GLT‐1 and system X_c_^−^,^27^, ^28^, ^29^, ^30^ resulting in the imbalance of glutamatergic neurotransmission and to the potentiation of withdrawal symptoms and drug relapse.^31^ Intriguingly, while the continuous morphine administration reduced the levels of extracellular glutamate in the NAc (glutamate spillover), the administration of secretome did not change the effect of continuous morphine treatment on glutamate extracellular level in this brain region. We did observe a significant increase in the slope of the no‐net‐flux glutamate determination induced by the secretome treatment, suggesting a potential effect on glutamate elimination dynamics, however, it did not significantly affect the expression of the glutamate transporters GLT‐1 or system X_c_^−^. A lack of changes in glutamatergic transporters was not expected, but can be explained as reports show that the alteration of the transporters levels depends on the specific opioid treatment scheme. For example, heroin self‐administration and withdrawal reduces the levels and activity of GLT‐1 and system X_c_^−^ and promotes an increase of glutamate spillover to the extracellular space in the NAc, opposite to the reduced spillover observed in the present study, an effect that also contributed to opioid dependence as the normalization of glutamate transporters levels reduced heroin reinstatement.^31^ On the other hand, GLT‐1 mRNA levels were reduced in the striatum of rats after 5 days of continuous morphine exposure via a subcutaneous pellet compared with placebo controls but were increased 2 h after naloxone‐induced withdrawal.^107^ Another study showed that hippocampal glutamate uptake was increased after 10 days of bidaily 10 mg/kg morphine administration, then initially reduced after 2 h of naloxone‐induced withdrawal, increased again after 12 h, and finally returned to baseline levels after 48 h.^70^ These results show that the requirement of the normalization of glutamatergic neurotransmission to reduce the withdrawal syndrome intensity is not completely understood. Likewise, in our previous report of the treatment with intranasal and intravenous hMSC secretome that significantly reduced morphine oral self‐administration, it did not alter GLT‐1 or xCT mRNA levels in the prefrontal cortex or nucleus accumbens compared to vehicle treatment, despite significant normalization of neuroinflammation and brain oxidative stress levels.^26^ Similarly, the lack of normalization of extracellular glutamate levels in the present model suggests that the hMSC secretome treatment reduces the opioid withdrawal syndrome through other mechanisms. The secretion of neurotrophic factors that could promote neurogenesis and induce neuro‐restoration has been associated with the therapeutic effects observed after the administration of secretome derived from MSCs in different animal models of neurologic diseases.^108^, ^109^ Further studies are needed to determine whether secretome administration could induce neuro‐restoration in this animal model and whether this phenomenon contributes to the observed therapeutic effects.

One limitation of our study is that we have not defined the full composition of the secretome derived from preconditioned MSCs. The secretome derived from MSCs is a complex mixture of hundreds of bioactive molecules, including proteins, lipids, and regulatory RNA, which could be released in a soluble form or inside small microvesicles called exosomes.^110^ Thus, identifying the molecules responsible for the observed therapeutic effects is a difficult task.^111^ Further studies are needed to evaluate the therapeutic potential of specific molecules present in the secretome and to study the dose–response profile of the secretome.

Finally, the observation that secretome administration also alleviates spontaneous withdrawal symptoms and reduces withdrawal scores following chronic voluntary oral morphine intake further strengthens the potential of the secretome as a therapeutic strategy for managing opioid withdrawal symptoms. The implications of this preclinical finding for the role of hMSC‐derived secretome in the treatment of morphine dependence and withdrawal warrant further exploration. It has been reported that MSCs show low immunogenicity, allowing for the allogenic transplantation of the cells or their secretome without the need for immunosuppression in the recipient.^112^ Additionally, adipose tissue‐derived MSCs can be easily obtained since adipose tissue is considered a waste product derived from cosmetic liposuction. Furthermore, MSC‐secretome can be lyophilized, facilitating its storage.^113^ Thus, MSC‐derived secretome could be envisioned as an off‐the‐shelf product for reducing opioid withdrawal syndrome in opioid‐addicted patients.

## 5 CONCLUSION

Overall, findings indicate the generation of therapeutic benefits by the simultaneous intravenous and intranasal administration of secretome derived from preconditioned hMSC for mitigating morphine‐induced withdrawal syndrome in two relevant animal models of morphine dependence. These effects were associated with a reduction of morphine‐induced neuroinflammation in the hippocampus and nucleus accumbens. While our study provides significant insights, further investigation is necessary to fully elucidate the mechanisms underlying the observed effects and to evaluate the therapeutic feasibility of secretome administration in other models of opiate abuse disorders.

## CONFLICT OF INTEREST STATEMENT

The authors declare no conflict of interest.

## Supporting information


Data S1.

## Data Availability

The data that support the findings of this study are available on request from the corresponding author. The data are not publicly available due to privacy or ethical restrictions.
