# Supplementary material for: Amelioration of morphine withdrawal syndrome by systemic and intranasal administration of mesenchymal stem cell‐derived secretome in preclinical models of morphine dependence
Source: CNS Neurosci Ther. 2023 Nov 6;30(4):e14517. doi: 10.1111/cns.14517 (PMC11017443; doi:10.1111/cns.14517)
Supplement: Supplementary file 1 — Data S1. [file CNS-30-e14517-s001.zip › Mauricio Quezada, CNS Neuroscience and Therapeutics Supplementary Figures.pdf]

Amelioration of morphine withdrawal syndrome by systemic and intranasal administration of mesenchymal stem cell-derived secretome in preclinical models of morphine dependence

Mauricio Quezada, Carolina Ponce, Pablo Berríos-Cárcamo, Daniela Santapau, Javiera Gallardo, Cristian De Gregorio, María Elena Quintanilla, Paola Morales, Marcelo Ezquer, Mario Herrera-Marschitz, Yedy Israel, Paula Andrés-Herrera, Lucia Hipólito, Fernando Ezquer

SUPPLEMENTARY FIGURE 1

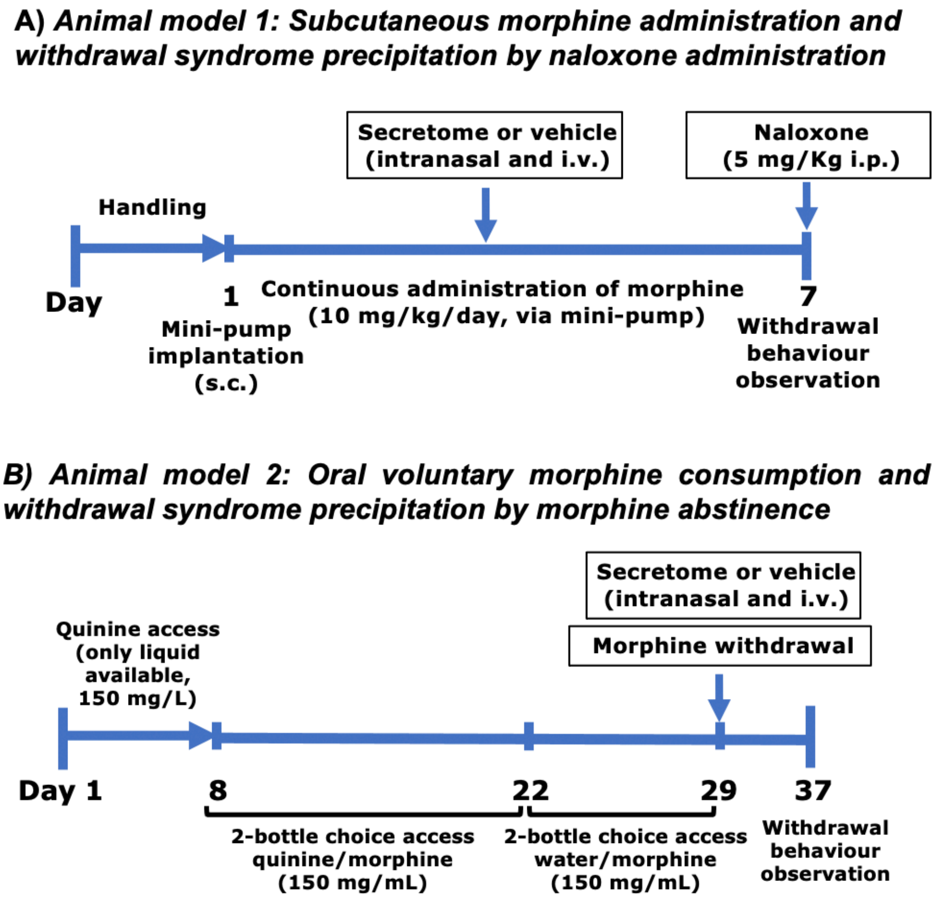

Supplementary Figure 1: Experimental Design. **(A)** *Animal model 1 Subcutaneous morphine administration and withdrawal syndrome precipitation by naloxone administration.* Female Wistar rats were implanted subcutaneously with osmotic mini-pumps releasing morphine at a dose of 10 mg/kg/day or saline. Three days post pump implantation, the morphine treated animals were

randomly divided into two groups. One group received simultaneously an intranasal (25µg proteins) and intravenous (25µg proteins) dose of secretome derived from $1x10^6$ preconditioned human MSCs, while the other group received the vehicle. Animals in the saline control group received the intranasal and intravenous administration of the vehicle. After seven days of continuous morphine exposure, rats were intraperitoneally injected with 5 mg/kg of the µ-opioid receptor antagonist naloxone to induce withdrawal. **(B)** Animal model 2. Oral voluntary morphine consumption and spontaneous withdrawal syndrome precipitation by morphine abstinence. Following an initial seven-day exposure to 0.15 mg/mL quinine hydrochloride as the only fluidic source, the animals transitioned to a two-week regimen where they were given a choice between two bottles, one contained quinine hydrochloride (0.15 mg/mL) and the other morphine sulfate (0.15 mg/mL). After this two-week period, the quinine bottle was removed, leaving the animals with a choice between tap water and morphine sulfate (0.15 mg/mL) for one additional week. A control group that had access only to water was also used. Withdrawal syndrome was spontaneously induced by discontinuation of morphine access after three weeks of morphine intake. On the same day, rats were administered simultaneously with an intranasal and intravenous dose of either secretome or saline. Somatic signs of morphine withdrawal were recorded for 30 minutes 48 hours later.

SUPPLEMENTARY FIGURE 2

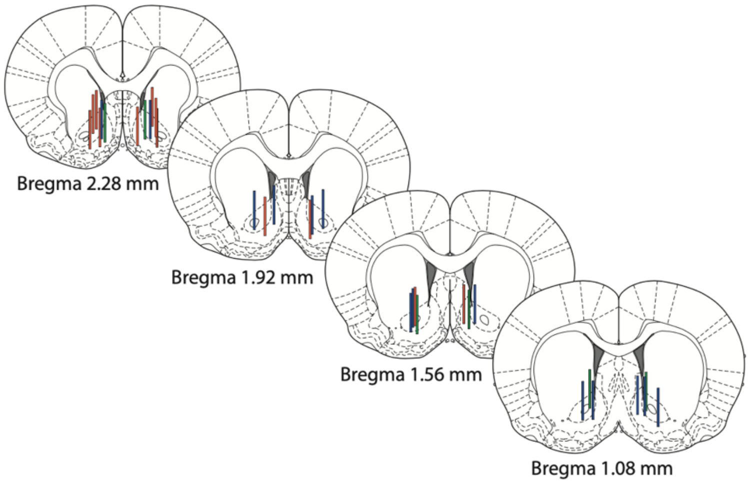

Supplementary Figure 2: Diagram of coronal sections indicating the placement of microdialysis probes. Illustrative scheme of the coronal sections showcasing the positioning of the microdialysis probes. All probe placements coincided with the areas marked by color-coded lines.

SUPPLEMENTARY FIGURE 3

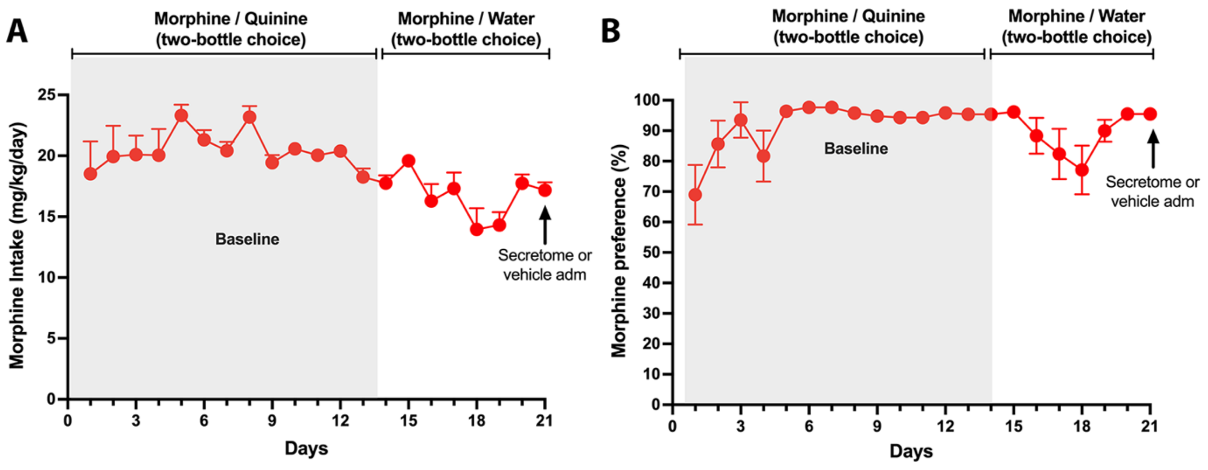

Supplementary Figure 3: Voluntary morphine intake by Wistar rats. One week after starting quinine intake, animals were exposed to 0.15 mg/mL quinine and 0.15 mg/mL morphine for two weeks using the two-bottle choice paradigm. Thereafter, animals were exposed to 0.15 mg/mL morphine and water (two-bottle choice) for one additional week. (**A**) Voluntary morphine intake is expressed as mg morphine consumed per kg of body weight per day. (**B**) Morphine preference (%). Data are expressed as mean ± SEM. *n* = 6. A control group (n=7) was given access to a bottle of water.

SUPPLEMENTARY FIGURE 4

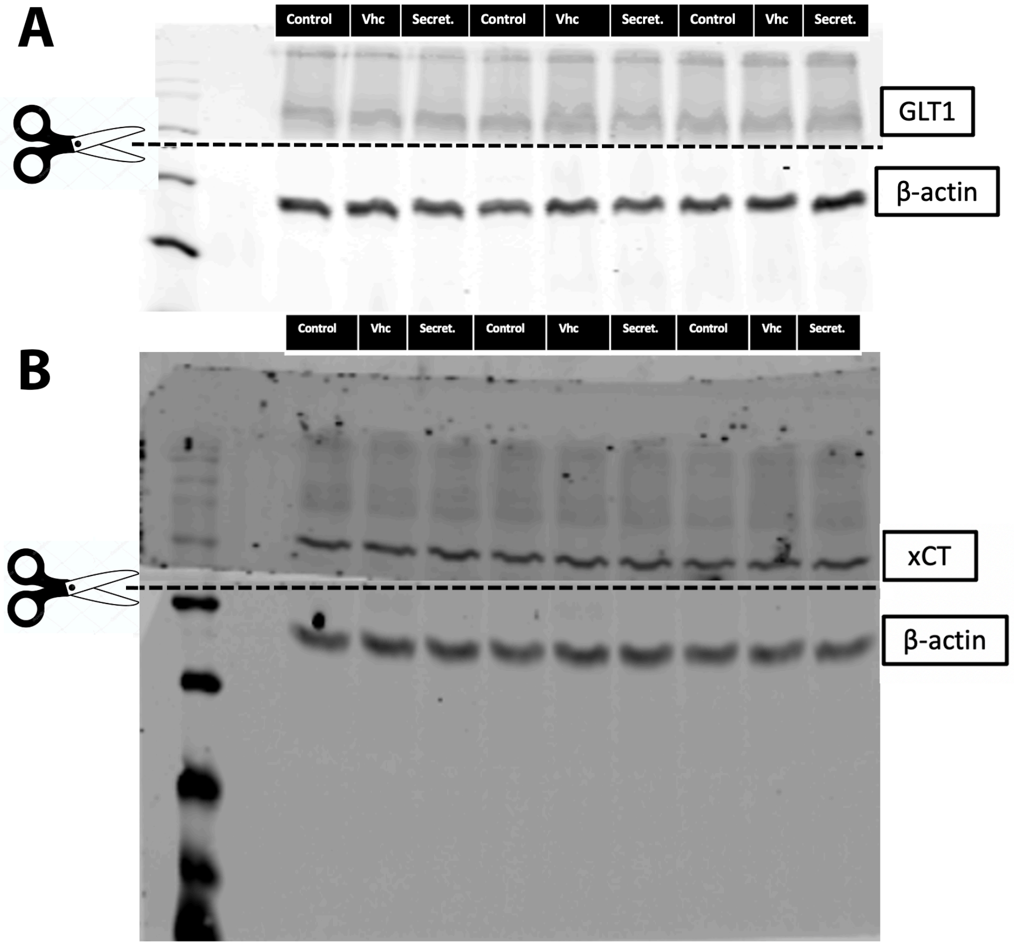

Supplementary Figure 4: Uncropped image of each cropped blot of Figure 7. (A) Representative image of Western Blot for the glutamate transporter GLT-1 and β-actin in NAc. **(B)** Representative image of Western Blot for the glutamate transporter xCT and β-actin in NAc.

SUPPLEMENTARY TABLE 1

Withdrawal Score		
Behavior	Numbers of events	Score
1.- Stretching	0	0
	1-24	1
	25-49	2
	50-79	3
	80 or more	4
2.- Jumps	0	0
	1-4	1
	5-9	2
	10-19	3
	20-39	4
	40 or more	5
3.- Wet dog shakes	If n > 2	2
4.- Ventral/dorsal flexes	Each n=2	1
5.- Forepaw tremors events	If n > 2	2
6.- Chewing	0	0
	1-4	1
	5-9	2
	10 or more	3
7.- Borrowings	0	0
	1-4	1
	5-9	2
	10-19	3
	20 or more	4
8.- Irritability	Scream when holding	3
9.- Percentage of the area covered by faeces	If % of area > 10	3
10.- Weight difference	—	Not included in the withdrawal score

Supplementary Table 1: Somatic withdrawal score assessment. The scoring system is graduated for the frequency of each parameter observed during a 30-minute evaluation period in a 5-litre beaker. This scoring system was adapted from previous studies [52,53].
