# Supplementary material for: Amelioration of morphine withdrawal syndrome by systemic and intranasal administration of mesenchymal stem cell‐derived secretome in preclinical models of morphine dependence
Source: CNS Neurosci Ther. 2023 Nov 6;30(4):e14517. doi: 10.1111/cns.14517 (PMC11017443; doi:10.1111/cns.14517)
Supplement: Supplementary file 1 — Data S1. [file CNS-30-e14517-s001.zip › Supplementary figure 4.docx]

**Supplementary Figure 4: Evaluation of secretome effects on GLT-1 and xCT glutamate transporter levels in the nucleus accumbens**. Representative uncropped images of Western Blot for the glutamate transporter GLT-1 (A) and xCT (B) in NAc for all experimental conditions. β-actin was used as a loading control.
